# Changes in accommodative function following small-incision lenticule extraction for high myopia

**DOI:** 10.1371/journal.pone.0244602

**Published:** 2020-12-30

**Authors:** Anders Gyldenkerne, Nicolaj Aagaard, Malene Jakobsen, Carina Toftelund, Jesper Hjortdal

**Affiliations:** Department of Ophthalmology, Aarhus University Hospital, Aarhus, Denmark; Singapore Eye Research Institute, SINGAPORE

## Abstract

**Purpose:**

To examine whether the amplitude of accommodation, the accommodative response, and the accommodative facility is affected and correlated with changes in higher-order aberrations for patients with high myopia surgically treated with small-incision lenticule extraction (SMILE).

**Methods:**

35 highly myopic eyes (myopic spherical equivalent of at least 6 diopters) of 35 patients treated with SMILE were included. Assessments were made before and 3 months after surgery. Donders push-up-method was used to measure the amplitude of accommodation. The accommodative response was assessed using an open-field autorefractor”Grand Seiko WAM-5500” (Grand Seiko Co. Ltd., Hiroshima, Japan) in combination with a Badal optometer and stimuli of accommodation at 0.0, 0.5, 1.25, 2.0, 3.0, and 4.0 D, respectively. Accommodative facility was measured at 40 cm with ±2,00D flipper lenses. All measurements of accommodation were performed monocularly with the refractive error corrected with soft contact lenses.

**Results:**

The amplitude of accommodation did not change statistically significantly (mean difference -0.24 D (SD 0.98), 95% CI of mean difference -0.58 D to 0.11 D, paired-sample *t*(34) = -1.39; *P* = 0.17). The accommodative responses at 0.0, 0.5, 1.25, 2.0, 3.0, and 4.0 D did not statistically significantly change either (*F*(6,29) = 1.15; *P* = .36). Finally, the accommodative facility was also unchanged with a mean difference of 1.11 cycles per minute (SD 5.11, 95% CI of mean difference -0.64 to 2.87, paired-sample *t*(34) = 1.29; *P* = 0.21). No clinically significant associations between changes in accommodation and higher-order aberrations were found.

**Conclusions:**

SMILE does not alter the amplitude of accommodation, the accommodative response, nor the accommodative facility for highly myopic patients, and the surgically induced corneal higher-order aberrations do not affect the accommodative function.

## Introduction

Small-incision lenticule extraction (SMILE) has been documented as a safe, predictable, and efficient surgical method for correcting myopia and astigmatism [1–4]. However, SMILE (and corneal refractive surgery in general) has been shown to increase higher-order aberrations such as coma and spherical aberration, consequently decreasing the quality of vision [5, 6].

Higher-order aberrations have been shown to change during accommodation, with spherical aberration generally considered to systematically change from positive to negative in contrast to changes in other higher-order aberrations, such as coma, that are less predictable [7–9]. Accommodation is known to be stimulated by blur, while cues for the directional information regarding accommodation include optical aberrations [10]. Higher-order aberrations (primarily spherical aberration) increase the depth of field and are thus known to influence the amplitude of accommodation [8]; through the use of adaptive optics, the accommodative response has also been shown to be influenced by spherical aberration and coma [9]. Following this line of thought, it is of interest whether induced higher-order aberrations following SMILE have an impact on accommodation.

To date, only few studies have examined how parameters of the accommodative function change due to corneal refractive surgery [11–13]. To the best of our knowledge, only a study by Zheng et al. [11] has evaluated accommodation in relation to SMILE for myopia; in their study, however, Zheng et al. only examined the accommodative response and did not report how the refractive error was corrected during the tests. The latter is particularly relevant given that the accommodative demand is higher for myopes when wearing contact lenses instead of spectacles [14]. Furthermore, it has been shown that the accommodative amplitude, the accommodative response, and the accommodative facility each describe different and independent aspects of accommodation [15, 16]; hence, all three parameters need to be measured to properly evaluate the accommodative function.

By correcting the sphere and astigmatism with contact lenses during the assessments of accommodation before and after surgery, this study investigated how SMILE for high myopia affected the accommodative function through the induction of higher-order aberrations. The accommodative function was assessed by measuring the amplitude of accommodation, the accommodative response, and the accommodative facility as recommended by several authors [16].

## Materials and methods

This prospective study included patients operated with SMILE for high myopia in the time period of May 2013 to June 2014. The study was performed in agreement with the tenets of the Declaration of Helsinki and was approved by the Danish Data Protection Agency and the Ethical committee of Central Region Denmark (case number 1-16-02-180-13). All patients provided informed written and verbal consent before surgery.

Patients included had the following characteristics: age 18 years to 35 years, stable refraction for at least 1 year, spherical correction from minus 6 diopters (D) to 10 D, astigmatism≤1 D, corrected distance visual acuity (CDVA) of at least 0.1 logarithm of the minimum angle of resolution (logMAR), and corrected near (40 cm) visual acuity (CNVA) of at least 0.5 logMAR [17]. Patients were excluded if any of the following criteria was present: ocular pathology affecting visual acuity or accommodation, use of medication influencing accommodation, an amplitude of accommodation<5 D or accommodative excess, and pregnancy or breastfeeding at the time of examination.

### Refractive evaluation

All measurements were performed before and 3 months after surgery by highly trained optometrists. Measurements included uncorrected distance visual acuity (UDVA), CDVA, subjective and cycloplegic spectacle refraction (obtained using tropicamide 10 mg/mL), autorefraction and autokeratometry (Tonoref II, Nidek Co. Ltd., Gamagori, Japan), Scheimpflug tomography including assessment of corneal higher-order aberrations (Pentacam HR, Oculus Optikgeräte GmbH, Wetzlar, Germany), and slitlamp evaluation and fundoscopy in mydriasis. The subjective refraction was measured at a vertex distance of 12 mm; the endpoint of the subjective refraction was the maximum plus that preserved best visual acuity. Distance visual acuity was measured using an electronic ETDRS chart at a distance of 6 metres with letter-by-letter scoring (each letter correctly guessed accounted for 0.02 logMAR); distance visual acuity measurements were terminated when at least 3 letters on a given line was not guessed correctly. Patients were required to stop wearing any soft contact lenses for at least 48 hours or any hard contact lenses for at least 2 weeks before examinations.

Total corneal higher-order aberrations (i.e. including both corneal surfaces) up to the 6^th^ order were measured for a 5.0 mm diameter zone under standard scotopic light settings and recorded using the Optical Society of America notation. The Zernike coefficient of spherical aberration was used directly for analyses. Total root mean square (RMS) of 3^rd^-order coma was calculated from the individual 3^rd^-order Zernike coefficient values of coma as

$$RMS(Total\;coma)=\sqrt{{(Z}_{3}^{1}{)}^{2}+{(Z}_{3}^{-1}{)}^{2}}$$

and residual higher-order aberrations were calculated by subtracting the Zernike coefficients of 4^th^-order spherical aberration and the 3^rd^-order coma terms from the total RMS of all higher-order aberrations (3^rd^ to 6^th^ order) as

$$RMS(Residual\;HOA)=\sqrt{{HOA}_{total}^{2}-(Z_{4}^{0}{)}^{2}-{(Z}_{3}^{1}{)}^{2}-{(Z}_{3}^{-1}{)}^{2}}$$

### Assessments of accommodation

At the preoperative examination, the patients’ dominant eye was recorded using the Miles test: patients were told to extend both arms and observe the distant ETDRS chart through a small hole formed with their hands with both eyes open; patients were then told to alternative open/close both eyes separately to establish which eye was dominant. Eye dominance was recorded because a previous study reported slightly different amplitudes of accommodation and accommodation facility for dominant vs. non-dominant eyes [18]. Because all patients were highly myopic, a significant difference in the accommodative demand would prevail depending on the correction used while performing tests of accommodation [14]. To ensure that accommodation was not affected by the use of spectacles, a soft contact lens (Dailies Total One, Alcon, Fort Worth, TX, USA) with a power equal to the subjective spherical equivalent of the individual eyes was worn throughout the tests of accommodation. Patients wore the contact lenses for 5 minutes before any further testing was performed. Then, to ensure that full emmetropia was completely reached, a trial lens was worn in addition to the contact lens. The trial lens prescription was determined by taking the average of 3 consecutive autorefractive measurements over the worn contact lens using an open-field autorefractor named Grand Seiko WAM-5500 (Grand Seiko Co. Ltd., Hiroshima, Japan) [19]; patients were told to focus on the smallest readable line on a distant vision chart while the autorefractive measurements over the contact lens were performed. In order to exclude any influence from vergence on the accommodation measurements, all tests were performed monocularly; moreover, all tests of accommodation were performed in the same room with the same lighting conditions (illuminance approximately equal to 170 lux).

The amplitude of accommodation was measured using Donders’ push-up method. Patients were instructed to focus on the line second from the bottom on a reduced vision chart and report when the letters became blurred as the chart was moved towards the eye from a distance. The amplitude of accommodation was recorded to the nearest 0.25 D.

**Fig 1:**
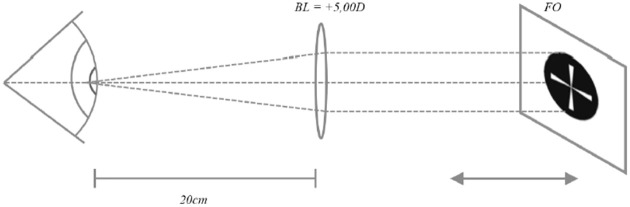
The Badal lens system used for measuring the accommodative response with constant angular size of the object. The +5.0 D Badal lens was placed 20 cm from the eye’s nodal point. A movable fixational object (FO) was used to stimulate accommodation at different vergences.

The accommodative response was measured using the Grand Seiko WAM-5500 open-field autorefractor combined with a +5.00 D Badal lens system [19, 20]; this setup allowed a movable fixation object to stimulate accommodation at different vergences with a constant angular size of the fixation object (Fig 1). Moving the target to distances of 20 cm, 18 cm, 16 cm, 14 cm, 12.5 cm, and 11.1 cm corresponded to accommodative stimuli of 0.0 D, 0.5 D, 1.25 D, 2.0 D, 3.0 D, and 4.0 D, respectively. At each stimulus, 5 autorefractive measurements were taken and the average was used for analysis. The order of which the accommodative stimuli were presented was randomized to avoid a consistent increase in the accommodative demand.

The accommodative facility was measured using a ±2.0 D flipper lens. Patients were instructed to first focus on a 0.5 logMAR letter line on a near vision chart at a distance of 40 cm and subsequently report as quickly as possible when the target was again in focus after viewing through first the +2.0 D lens and then the -2.0 D lens as the examiner flipped the lens when the target was reported clear. One cycle constituted focusing the target through both lenses, and the number of cycles completed in one minute was recorded as the accommodative facility; this test was performed as the final part of the examination to avoid accommodative fatigue influencing the other accommodative assessments.

### Surgical technique

The surgical technique has been explained in detail elsewhere [2]. Briefly, a 500 kHz Visumax femtosecond laser (Carl Zeiss Meditec AG, Jena, Germany) was used; cap thickness ranged from 100 to 130 μm, cap diameter from 7.3 to 7.9 mm, and lenticule diameter from 6.0 to 7.0 mm with a transition zone of 0.0 to 0.1 mm. The laser cut energy index was between 25 and 34 and the spot spacing between 2.5 μm and 4.5 μm. Lenticule dissection was done with a blunt spatula, and the lenticule was extracted through a 30–40° opening at 12 o’clock. Patients were postoperative treated with tobramycin-dexamethasone q.i.d. tapered over 2 weeks.

### Statistics

Statistical analysis of the dominant eye of each patient was performed in Stata (version 15.0, Stata Corp, College Station, TX, USA). Data was assessed for normality using the Shapiro-Wilk normality test and through inspection of histograms and QQ-plots. Paired t-tests were applied for pairwise comparisons, and a multivariate repeated measurements model was used to assess the accommodative stimulus-response curves’ equality. Pearson correlation coefficients and simple linear regression models were used to compare changes in accommodative functions and surgically induced changes in spherical aberration, total coma, and residual higher-order aberrations; the validity of regression models was assessed through inspection of diagnostic plots of the residuals. A *P*-value below 0.05 was considered statistically significant.

Sample size calculations were performed for each accommodative parameter investigated in this study with power set to 90% and the level of significance set to 0.05: For the amplitude of accommodation, using a standard deviation of 2.79 D for paired measurements [21], a sample size of 39 eyes would be needed to find a difference of 1.5 D [22]; for the accommodative response, using a standard deviation of 0.40 D for paired measurements for a stimulus of 2.50 D [23], a sample size of 29 eyes would be needed to find a difference of 0.25 D; finally, for the accommodative facility, using a standard deviation of 1.0 cpm for paired measurements [24], a sample size of 13 eyes would be needed to find a difference of 1.0 cpm.

## Results

The study included 35 eyes of 35 patients; initially, 43 patients accepted participance in the study, but 7 patients did not return for the postoperative visit and 1 patient became pregnant after surgery.

Table 1 shows the patient demographics. Mean age was 27.9 years (SD 4.0), and mostly right eyes were dominant (25 out of 35). The postoperative spherical equivalent was close to 0 (mean -0.21 D; [SD 0.42]) with 77.1% of patients being within ±0.50 D of emmetropia and 97.1% being within ±1.0 D. No patients lost more than 1 line of CDVA.

**Table 1:** Demographic data.

Parameter	Preoperative	Postoperative
	Mean (SD)	Mean (SD)
	[range]	[range]
Sex (M/F)	20/15	
Age [years]	27.9 (4.0) [21 to 35]; median: 28	
Dominant eye (R/L)	25/10	
UDVA [logMAR]		0.02 (0.12)
		[-0.2 to 0.36]
UDVA ≤ 0.1 logMAR, n; %		28/35; 80.0%
CDVA (logMAR)	-0.064 (0.06)	-0.061 (0.06)
	[-0.18 to 0.04]	[-0.2 to 0.1]
Change in CDVA, n; %		
• Lost 2 lines		0/35; 0.0%
• Lost 1 line		8/35; 22.9%
• No change		20/35; 57.1%
• Gained 1 line		7/35; 20.0%
• Gained 2 lines		0/35; 0.0%
Sphere (D)	-7.02 (0.93)	-0.02 (0.40)
	[-9.0 to -5.75]	[-1.0 to 0.75]
Cylinder (D)	-0.49 (0.35)	-0.38 (0.34)
	[-1.0 to 0.0]	[-1.0 to 0.0]
Spherical equivalent (D)	-7.27 (0.90)	-0.21 (0.42)
	[-9.0 to -5.88]	[-1.13 to 0.75]
Spherical equivalent within ± 0.50 D, n; %		27/35; 77.1%
Spherical equivalent within ± 1.0 D, n; %		34/35; 97.1%

UDVA = uncorrected distance visual acuity; CDVA = corrected distance visual acuity; D = diopter; logMAR = logarithm of the minimum angle of resolution; SD = standard deviation

Table 2 shows the results for assessment of accommodation. Importantly, none of the investigated parameters showed statistically significant changes due to surgery.

**Table 2:** Assessment of accommodation.

Accomodative parameter	Before surgery	After surgery	Difference*	95% CI of mean difference	Test results
	Mean (SD)	Mean (SD)	Mean (SD)		
Amplitude (D)	8.37 (1.68)	8.13 (1.52)	-0.24 (0.98)	-0.58 to 0.11	Paired t(34) = -1.39; *P* = .17
Response at each stimulus (D):					
• Stimulus 0 D:	0.16 (0.32)	0.18 (0.24)	0.023 (0.40)	-0.12 to 0.16	
• Stimulus 0.5 D:	0.37 (0.25)	0.31 (0.24)	-0.056 (0.33)	-0.17 to 0.06	
• Stimulus 1.25 D:	0.62 (0.22)	0.58 (0.23)	-0.05 (0.28)	-0.14 to 0.05	*F*(6,29) = 1.15;
• Stimulus 2.0 D:	1.39 (0.30)	1.29 (0.30)	-0.10 (0.32)	-0.22 to 0.01	*P* = .36†
• Stimulus 3.0 D:	2.24 (0.39)	2.13 (0.51)	-0.10 (0.45)	-0.26 to 0.05	
• Stimulus 4.0 D:	3.17 (0.45)	3.19 (0.60)	0.02 (0.45)	-0.14 to 0.18	
Facility (cpm)	9.14 (6.20)	10.26 (6.40)	1.11 (5.11)	-0.64 to 2.87	Paired t(34) = 1.29; *P* = .21

SD = standard deviation; CI = confidence interval; D = diopter; cpm = cycles per minute.

*:”difference” refers to postoperative values minus preoperative values.

^†^: Multivariate repeated measurements test for null-hypothesis that all values are equal to zero

**Fig 2:**
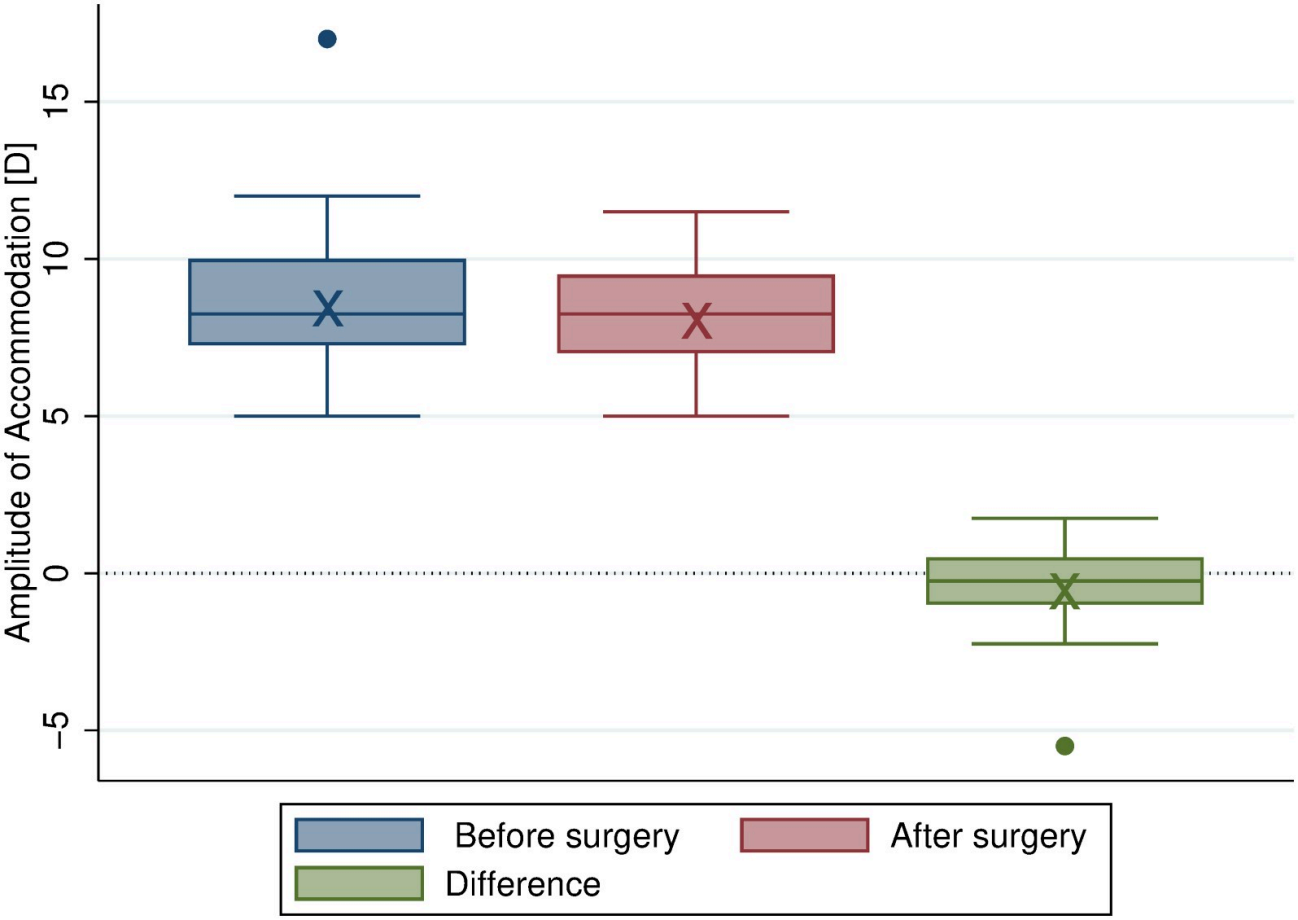
Box plots of the amplitude of accommodation showing no significant change due to surgery. The box indicates the interquartile range, the middle line within the box indicates the median, and the whiskers display the adjacent values (i.e. 1.5 times the interquartile range beyond the 25^th^ and 75^th^ percentile). Also, the “x” marks the mean. Red box: Before surgery. Blue box: After surgery. Green box: Surgically induced difference (not statistically significant, *P* = .17).

Fig 2 shows box plots of the amplitude of accommodation before and after surgery; the postoperative amplitude of accommodation was on average about 0.25 D lower than the preoperative amplitude of accommodation.

**Fig 3:**
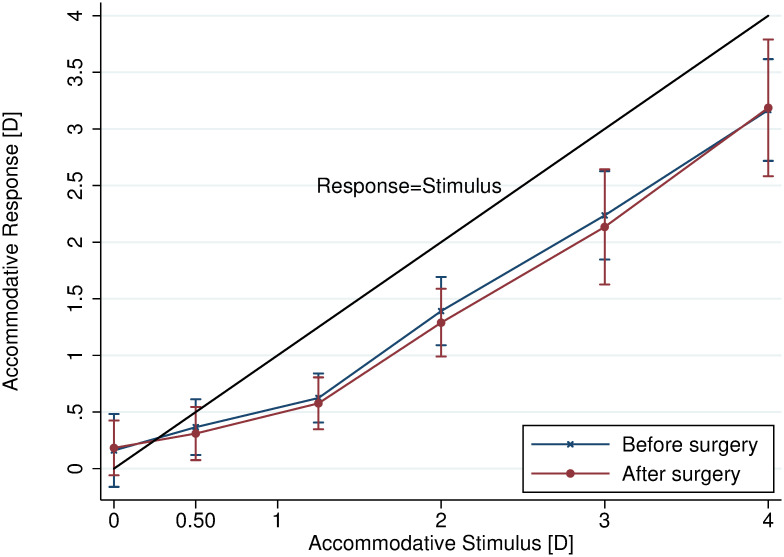
Practically equal accommodative response-stimulus curves before and after surgery. Accommodative lead is seen for 0 D and increasing lag is seen with increasing stimulus. Error bars represent ± 1 standard deviation. Function y = x shown for comparison.

Fig 3 shows the accommodative response before and after surgery with a reference line for comparing with the ideal response (response equal to stimulus). The characteristic combination of an initial non-linear segment accompanied by an initial accommodative lead at low stimuli followed by a linear tendency with increasing accommodative lag at larger stimuli was noticed.

**Fig 4:**
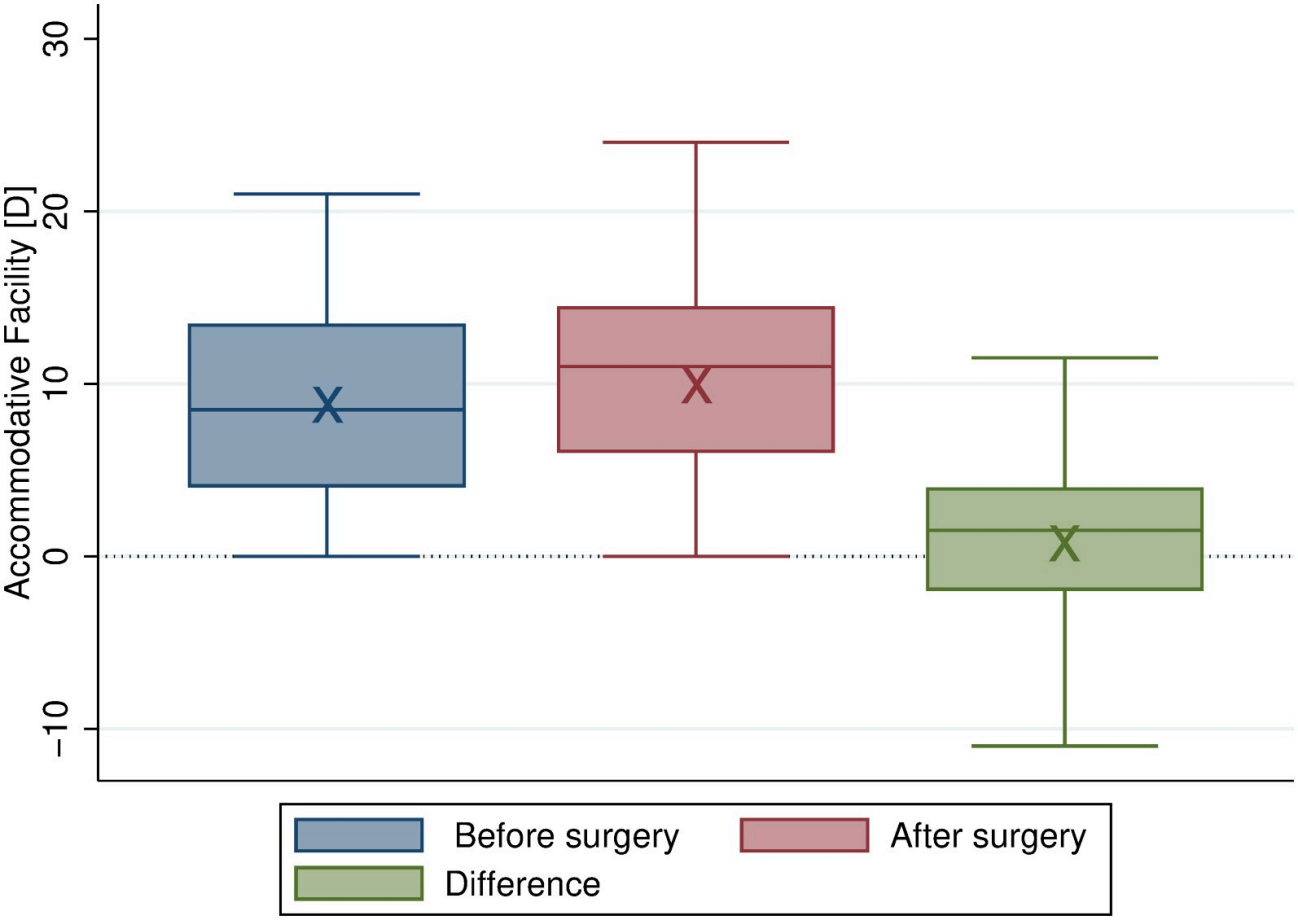
Box plots of the accommodative facility showing no significant change due to surgery. The box indicates the interquartile range, the middle line within the box indicates the median, and the whiskers display the adjacent values (i.e. 1.5 times the interquartile range beyond the 25^th^ and 75^th^ percentile). Also, the “x” marks the mean. Red box: Before surgery. Blue box: After surgery. Green box: Surgically induced difference (not statistically significant, *P* = .21).

Fig 4 shows box plots of the accommodative facility before and after surgery. The spread of data was practically unchanged following surgery with differences centering around 0 with an average difference of about 1 cpm.

Corneal higher-order aberrations changed due to surgery: spherical aberration and total coma showed statistically changes after surgery, whereas the residual higher-order aberrations did not. Firstly, spherical aberration decreased slightly from the preoperative value of 0.070 μm (SD 0.05) to a postoperative value of 0.013 μm (SD 0.08), yielding a mean change of -0.057 μm (SD 0.07) (95% CI -0.08 μm to -0.03 μm; paired t(34) = 3.21; *P* < .001). Secondly, total coma increased from 0.13 μm (SD 0.07) to 0.21 μm (SD 0.14) with a mean change of 0.077 μm (SD 0.14) (95% CI 0.028–0.13 μm; paired t(34) = -4.60; *P* = .003). Lastly, residual higher-order aberrations increased from 0.014 μm (SD 0.09) to 0.18 μm (SD 0.07) with a mean change of 0.042 μm (SD 0.12) (95% CI -0.0015 to 0.085 μm; paired t(34) = 1.97; *P* = .06).

Linear regression analysis with the changes in each of the observed differences in accommodative assessments on age, change in spherical equivalent, change in spherical aberration, and change in total coma was performed; none of these regressions revealed any significant statistical nor clinical relationships (S1 Appendix). Likewise, Pearson correlations between the changes in accommodation and higher-order aberrations were insignificant.

Finally, the same analyses on accommodation performed on the dominant eyes were also performed on the non-dominant eyes with equivalent conclusions.

## Discussion

The refractive and visual acuity outcomes in this study agree well with other studies on SMILE for high myopia [1–4].

The amplitude of accommodation did not change statistically significantly after SMILE. The postoperative values were on average slightly lower (about 0.25 D), but considering the 95% confidence interval (-0.58 to 0.11) it seems safe to say that the accommodative amplitude was not affected by the surgery. The observed standard deviation on the differences (0.98 D) is in accordance with what has been reported by other studies for the push-up method [16, 21, 22, 25]. Had we used the minus-lens or the push-down method for measuring the amplitude of accommodation, different absolute results would have been expected [16, 21]; however, as this study focused on differences rather than absolute values, we consider this a minor concern. Similarly, the push-up method used here did not correct for increasing image size as the target came closer—nonetheless, since the focus was on change and not absolute measurements, this should not affect the conclusions. To our knowledge, no other study has yet examined the amplitude of accommodation following SMILE; Liu et al. [13] found no change in amplitude of accommodation following laser-assisted in situ keratomileusis for mild to moderate myopia (method used for measuring the amplitude of accommodation not reported), while Karimian et al. [12] found a slight statistically significant increase of about 0.6 D for patients aged under 30 but no difference for patients aged above 30 following photorefractive keratectomy (PRK) using the minus-lens method. Given that Rosenfield et al. [22] proposed that a clinically significant change of at least ±1.50 D should be adopted as a minimum significant shift in amplitude of accommodation, the change of 0.60 D for young patients found by Karimian et al. [12] also seems negligible.

The accommodative response did also not show any statistically significant changes for the stimuli tested. Measurements of accommodative response with the Grand Seiko WAM-5500 has been validated in previous studies [19, 26]; our data showed that the standard deviation of the differences tended to increase as the stimulus for accommodation increased, which was thus likely due to increased variability in the subjects’ accommodative response for larger stimuli. The only other study on accommodative change after SMILE (that we are aware of) by Zheng et al. [11] measured the static accommodative response on patients with mean age 23.34 years (SD 2.90) treated for moderate to high myopia. Zheng et al. [11] did not report how patients’ refractive error was corrected during measurements; however, they also used the Grand Seiko WAM-5500 for stimuli from 2.0 to 5.0 D and found a statistically significantly smaller accommodative lag and correspondingly larger response after surgery with an average difference of 0.22 D. Albeit the results by Zheng et al. are in contrast to our study where no significant differences were found, it is reassuring that SMILE does not seem to have detrimental effects on the accommodative response.

The accommodative facility, the last of the assessments of accommodation tested in this study, increased about 1 cpm following surgery, though not statistically significantly. The observed standard deviations for the accommodative facility in this study were about 5–6 cpm, which is high but still in agreement with what has been reported previously [12, 16, 27]. For comparison, Adler et al. [27] reported standard deviations of 3.8 cpm for schoolchildren aged 4–12 (average 8.1 years [SD 2.1]) and concluded that the test should be repeated if the initial result proved low; the standard deviation has been reported as even higher for adults using ± 2.0 D lenses for monocular testing (typically ± 5 cpm) [16]. Karimian et al. [12] found a significant increase in accommodative facility 3 months after PRK for patients aged below as well as above 30 years; the standard deviations reported in their study were very similar to what was seen in our study, but their preoperative values were lower (approx. 5 for patients aged above 30 and 7 for patients below 30 years of age) while the 3-months results were quite similar to ours with mean values of about 10–11 cpm. Judging from this study and the paper by Karimian et al., there does not seem to be a decrease in accommodative facility following refractive surgery [12].

In summary, none of the accommodative assessments performed in this study worsened following SMILE for high myopia; this result agrees with the relatively few other studies published on the topic [11–13]. The methodology in this study, however, was slightly different compared to those previous studies in that refraction errors in the present study were corrected with contact lenses to the greatest extent possible at both time visits, removing any influence from spectacles vs. contact lenses and any residual refraction on the assessments of accommodation.

Higher-order aberrations increased significantly in an amount very similar to what has been reported previously on SMILE for high myopia over a 5 mm pupil [28]. We found a decrease in spherical aberration of 0.057 μm (SD 0.07), an increase in coma of 0.077 μm (SD 0.14), and an increase in residual higher-order aberrations of 0.042 μm (SD 0.12). Although these changes were relatively small, experimentally induced changes as low as 0.05 μm has been reported capable of affecting visual acuity depending on the particular combination of wavefront errors [6]. Theoretical studies examining the effect of higher-order aberrations on accommodation by the use of adaptive optics have shown that changes in both spherical aberration and coma can influence accommodation; however, the experimentally induced changes in higher-order aberrations used to demonstrate these effects were as high as 1–2 μm, significantly larger than the mean increase seen in this study [8, 9]. Because the lower-order aberrations sphere and astigmatism were fully corrected during the tests of accommodation both before and after surgery, the essential difference between the pre- and postoperative conditions in this study was the level of higher-order aberrations; the relatively small amount of induced higher-order aberrations did not affect the assessments of accommodation to any significant degree. Thus, the induced higher-order aberrations following SMILE for high myopia did not significantly affect accommodation for patients in this study.

Linear regression analysis did not show any significant relations between changes in accommodative parameters and surgically induced changes in spherical equivalent, higher-order aberrations, or age at surgery. This is reassuring, indicating that patients’ age and preoperative refraction does not impact postoperative accommodation (given that correction with contact lenses is used to measure accommodation before and after surgery). Also, supplementing the discussion in the paragraph above, the induced higher-order aberrations did not prove to be substantial. Finally, the conclusions did not depend on whether the dominant or non-dominant eye was chosen for analysis [18].

This study has limitations. The sample size was relatively small; unfortunately, 7 patients did not return for the postoperative measurements (we could find no indication as to why they did not return). The accommodative facility proved more variable than anticipated, suggesting that future sample size calculations should implement a larger standard deviation than what was used in the present study. Further studies with larger sample sizes would be desirable in order to corroborate this study’s findings. Also, the induction of higher-order aberrations was measured with a corneal tomographer—what is visually relevant is of course not the corneal aberrations per se but the ocular aberrations; given that changes were analyzed, however, it would be unlikely that conclusions would have been different had an ocular wavefront analyzer been used. Finally, one could imagine that measuring e.g. the amplitude of accommodation and the accommodative facility several times in a row and using the average could have resulted in lower observed standard deviations; however, one would particularly with the accommodative facility test risk that patients would be fatigued resulting in increasingly lower results with the test.

In conclusion, this study did not find that SMILE for high myopia had any statistically nor clinically significant effects on the amplitude of accommodation, the accommodative response, or the accommodative facility. The preoperative assessments of accommodation were performed with patients wearing contact lenses to correct their refractive error, meaning that any potential accommodative problems corresponding to a shift from a correction in the spectacle plane to the corneal plane were neutralized; based on the results of this study, patients with high myopia do not need to be informed about any particular risks for reduced accommodation due to the surgical procedure of SMILE per se, but should of course (as for all patients with high myopia) be informed about the extra accommodative effort needed when changing correction from the spectacle plane to the corneal plane.

## Supporting information

S1 File Anonymized dataset. (XLS)

S1 Appendix Regressions of accommodative function on refractive parameters. (PDF)
