# Supplementary material for: Changes in accommodative function following small-incision lenticule extraction for high myopia
Source: PLoS One. 2020 Dec 30;15(12):e0244602. doi: 10.1371/journal.pone.0244602 (PMC7773189; doi:10.1371/journal.pone.0244602)
Supplement: S1 Appendix — (PDF) [file pone.0244602.s002.pdf]

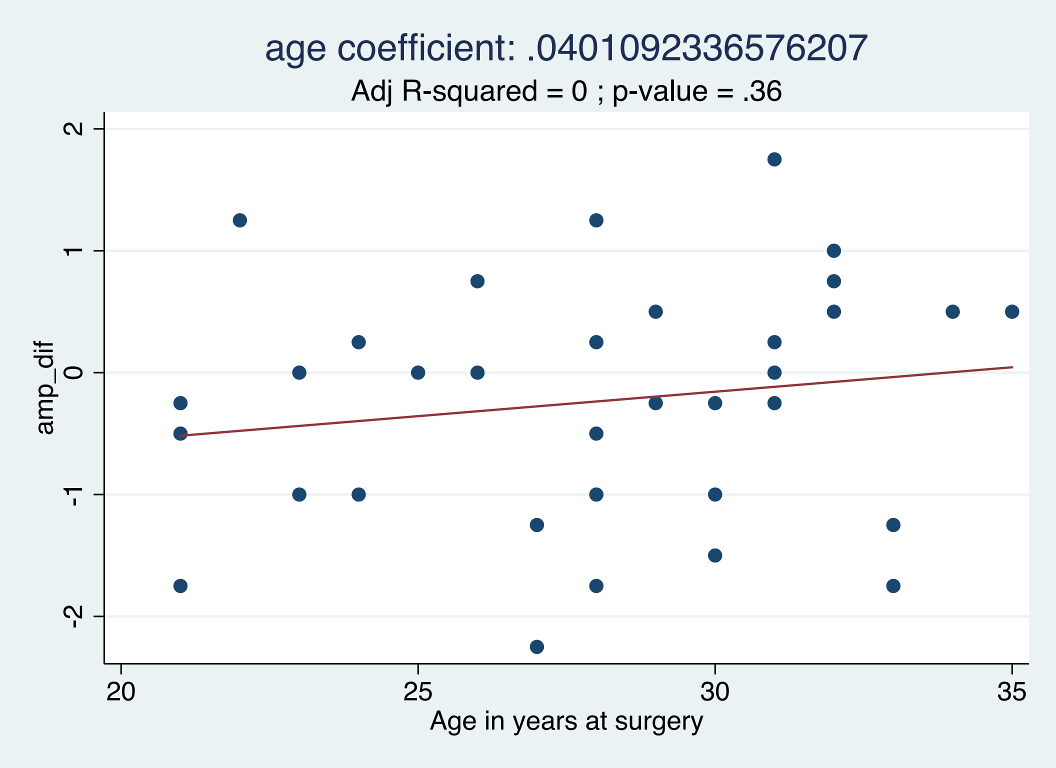
age coefficient: .0401092336576207
Adj R-squared = 0 ; p-value = .36
amp_dif
2
1
0
-1
-2
20
25
30
35
Age in years at surgery

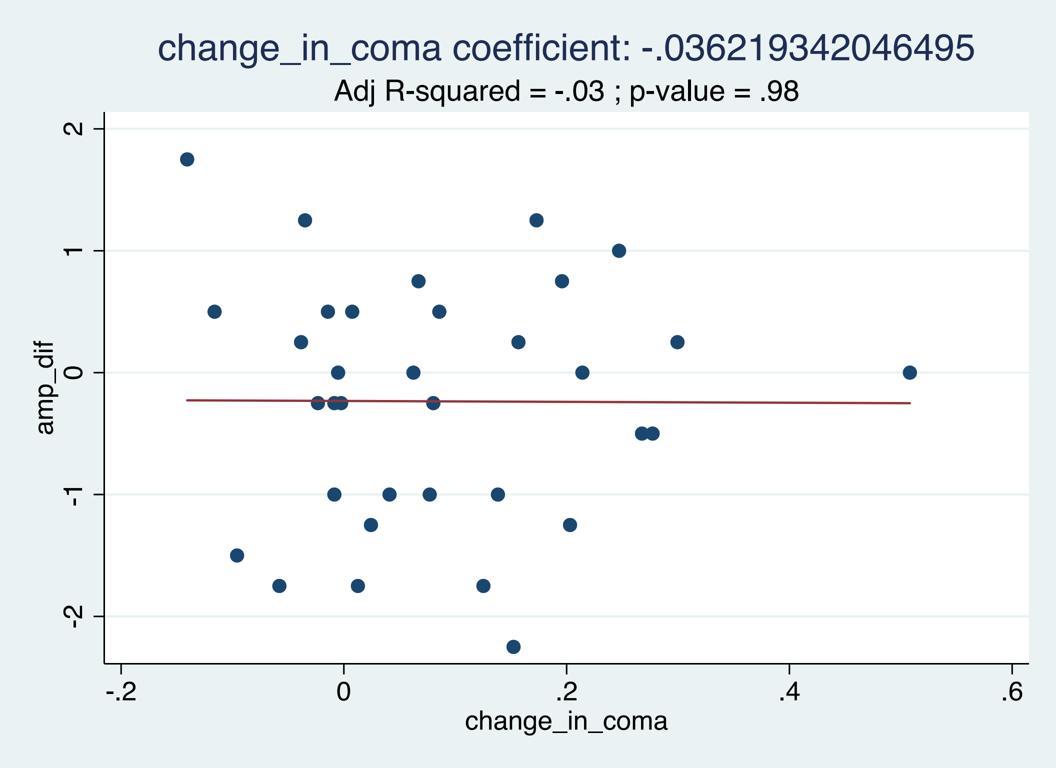
change_in_coma coefficient: -.036219342046495
Adj R-squared = -.03 ; p-value = .98
amp_dif
2
1
0
-1
-2
-.2
0
.2
.4
.6
change_in_coma

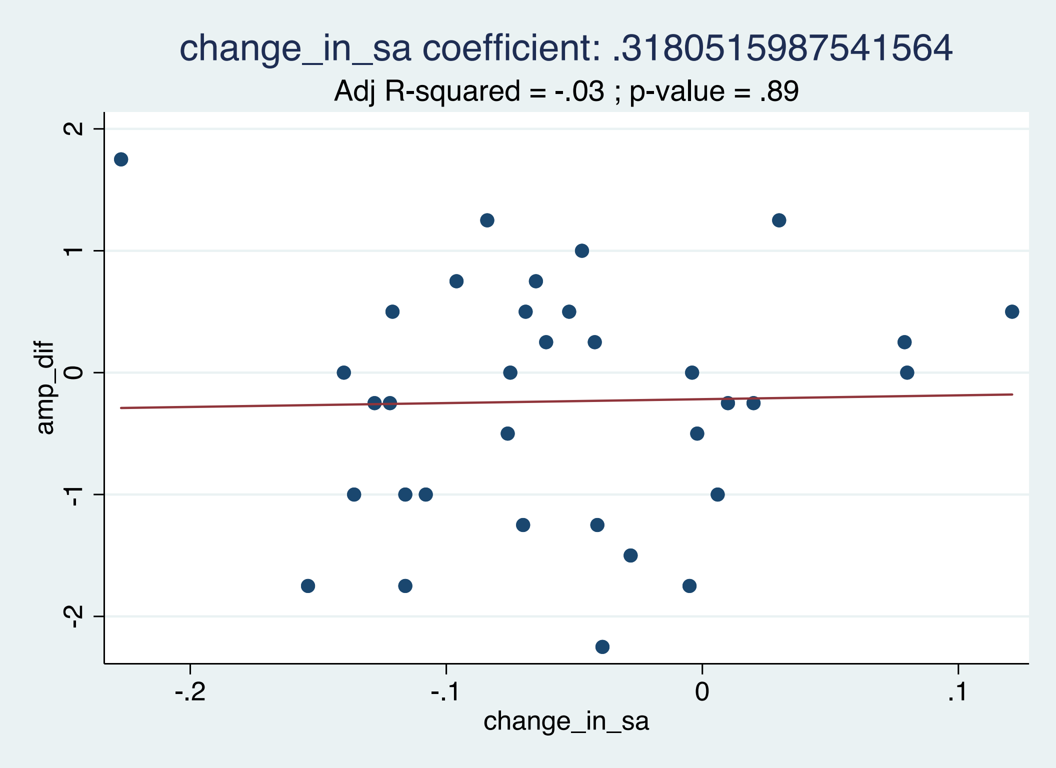

change_in_sa coefficient: .3180515987541564
Adj R-squared = -.03 ; p-value = .89
amp_dif
2
1
0
-1
-2
-.2
-.1
0
.1
change_in_sa

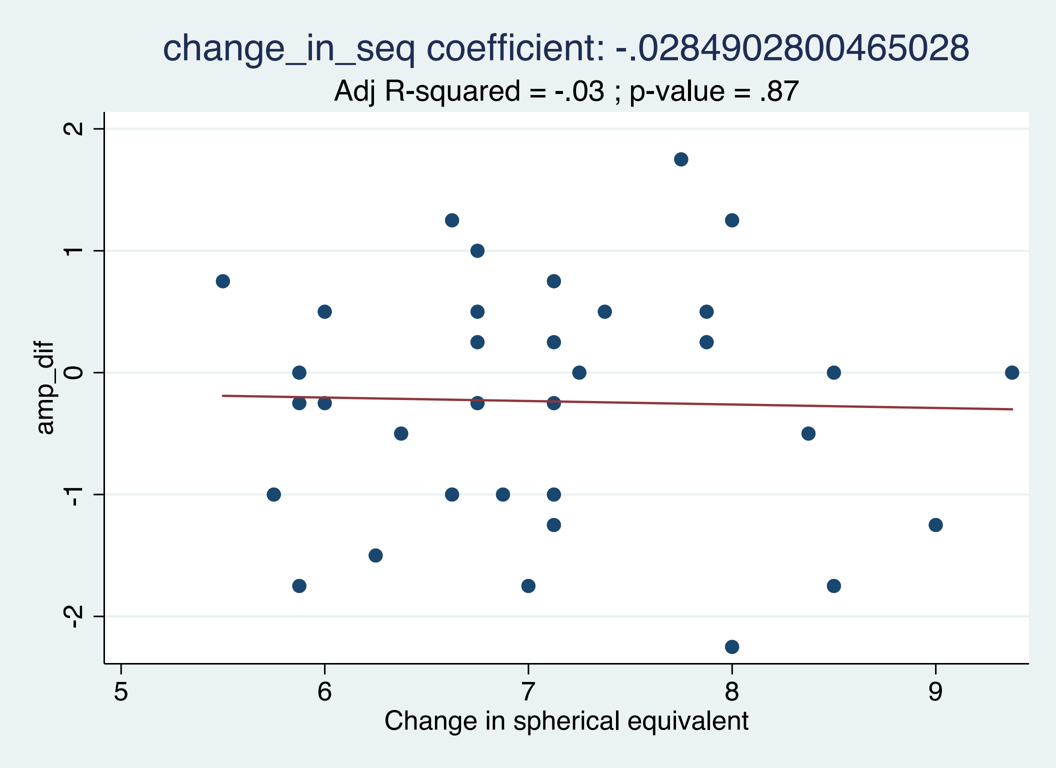
change_in_seq coefficient: -.0284902800465028
Adj R-squared = -.03 ; p-value = .87
amp_dif
2
1
0
-1
-2
5
6
7
8
9
Change in spherical equivalent

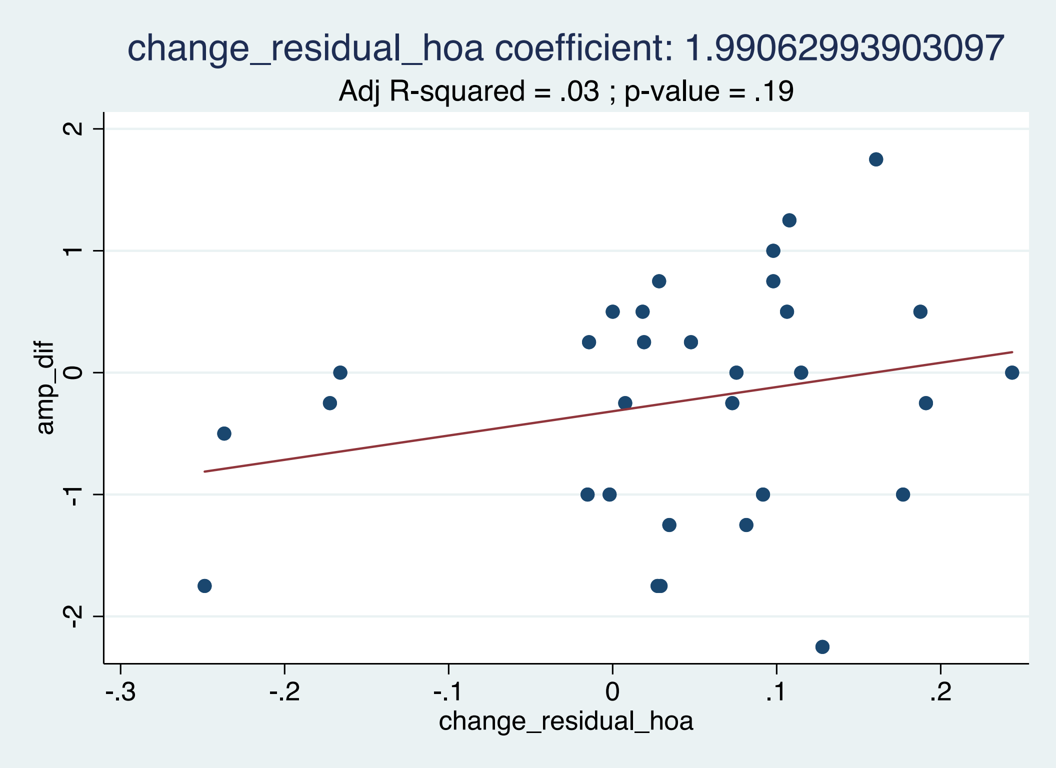
change_residual_hoa coefficient: 1.99062993903097
Adj R-squared = .03 ; p-value = .19
amp_dif
2
1
0
-1
-2
-.3
-.2
-.1
0
.1
.2
change_residual_hoa

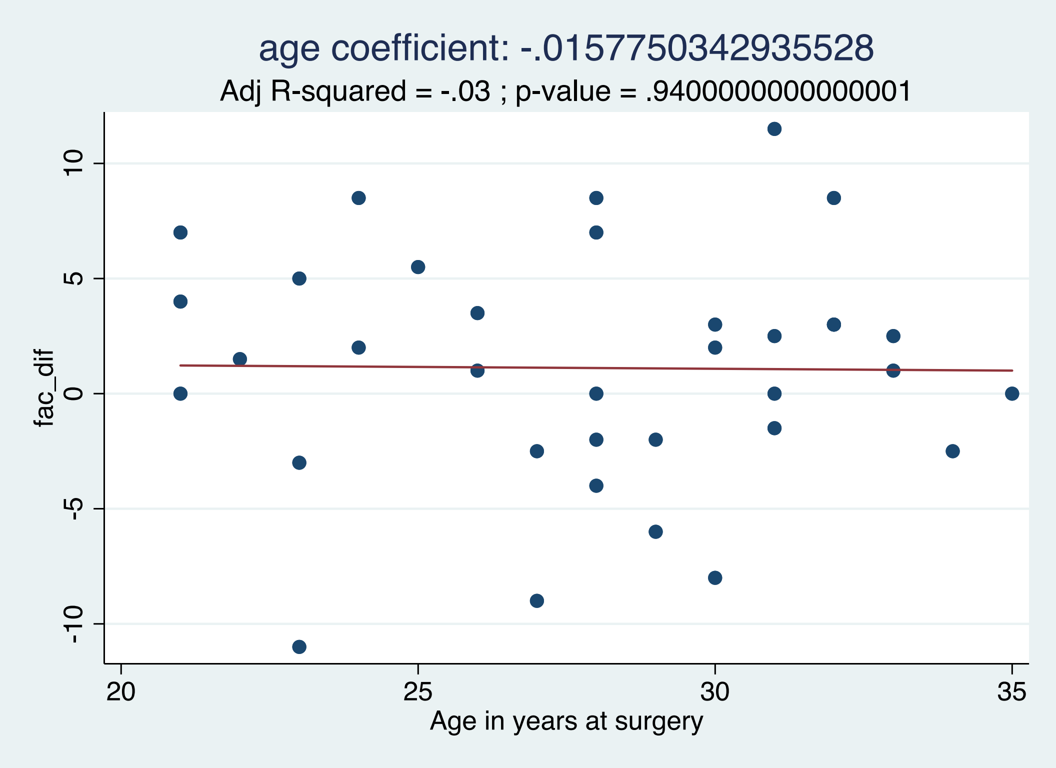
age coefficient: -.0157750342935528
Adj R-squared = -.03 ; p-value = .9400000000000001
fac_dif
-10
-5
0
5
10
20
25
30
35
Age in years at surgery

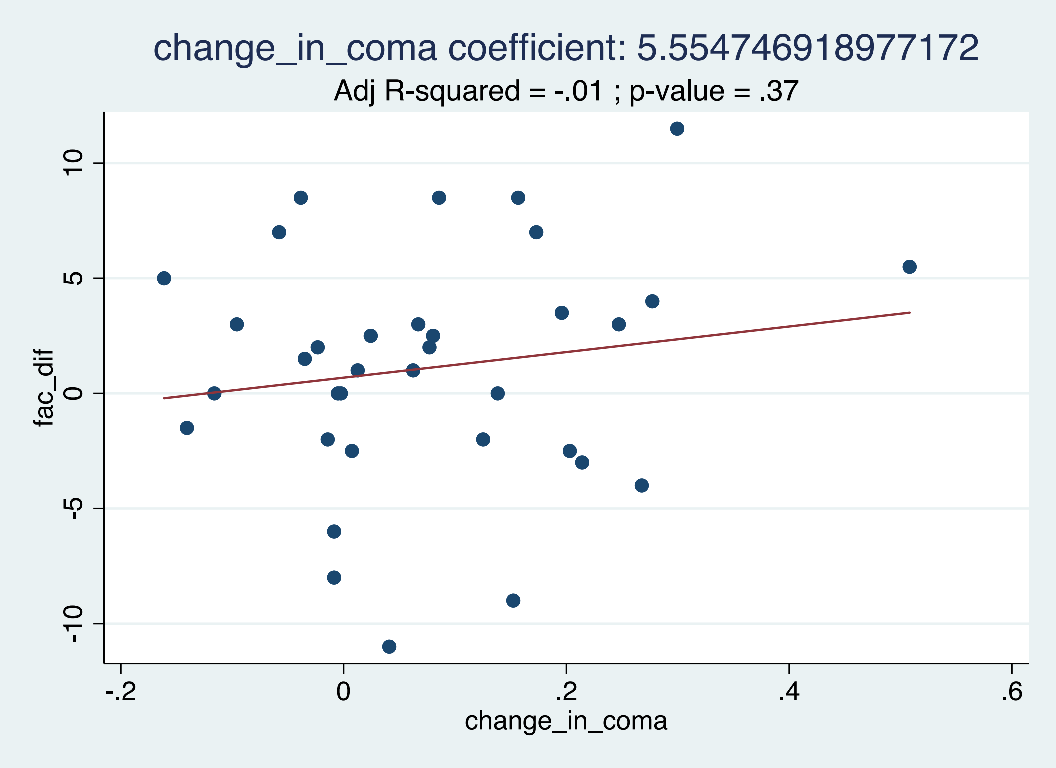

change_in_coma coefficient: 5.554746918977172
Adj R-squared = -.01 ; p-value = .37
fac_dif
10
5
0
-5
-10
-.2
0
.2
.4
.6
change_in_coma

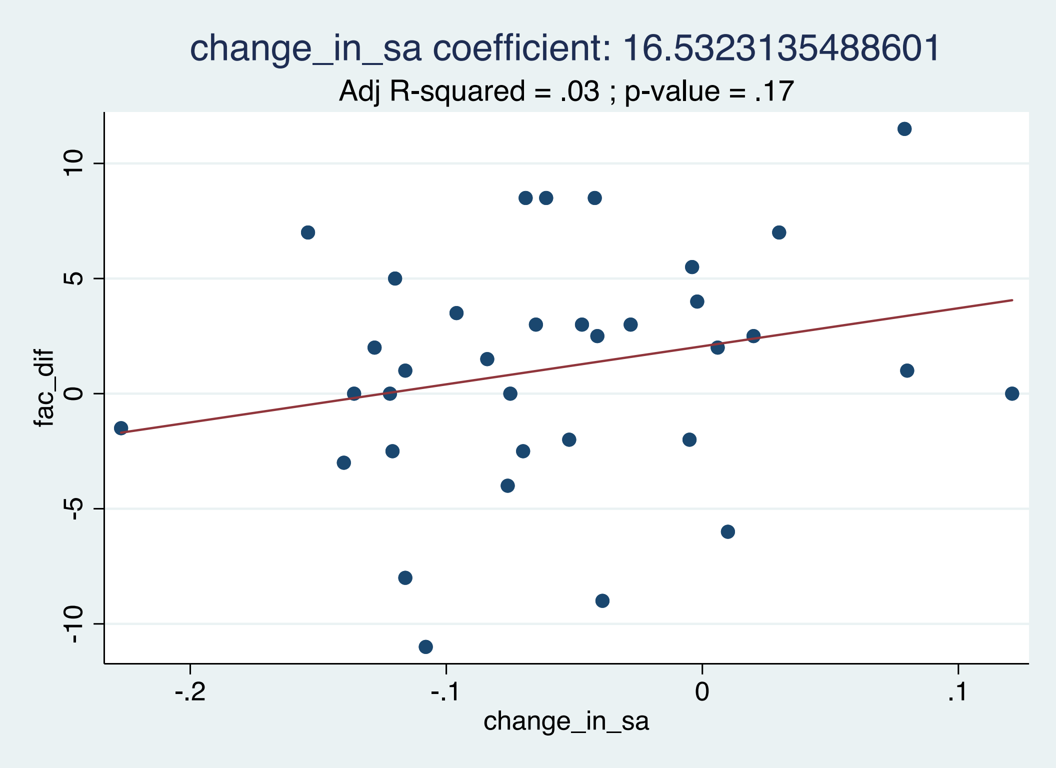
change_in_sa coefficient: 16.5323135488601
Adj R-squared = .03 ; p-value = .17
fac_dif
10
5
0
-5
-10
-.2
-.1
0
.1
change_in_sa

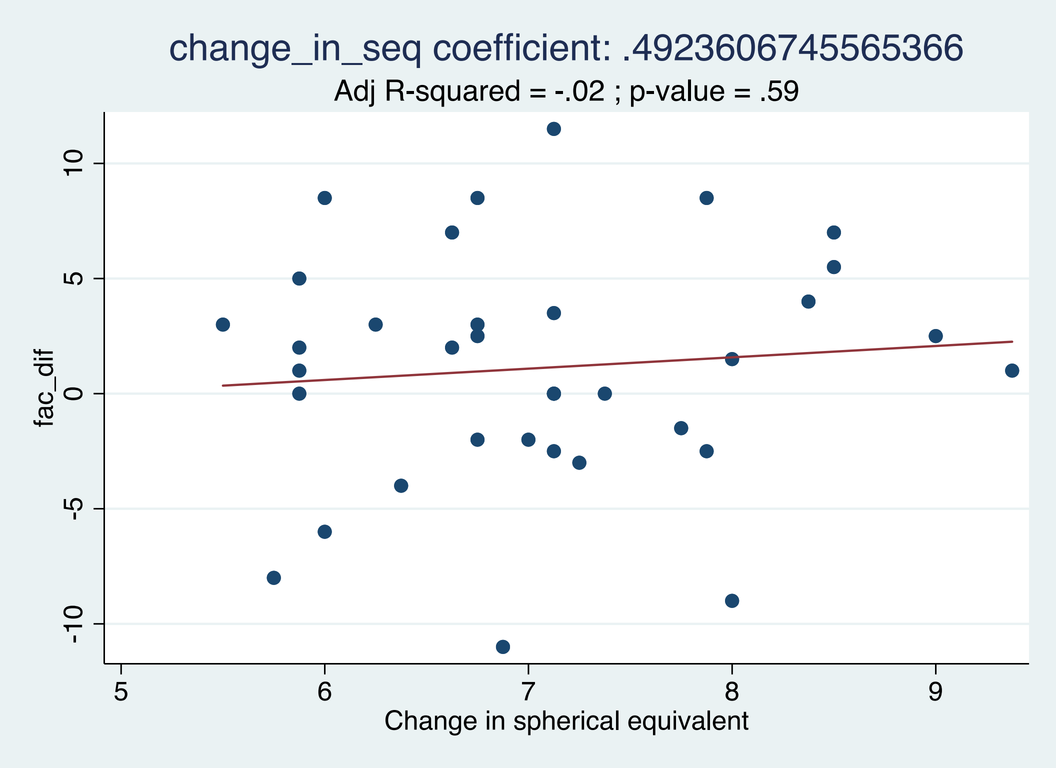
change_in_seq coefficient: .4923606745565366
Adj R-squared = -.02 ; p-value = .59
fac_dif
10
5
0
-5
-10
5
6
7
8
9
Change in spherical equivalent

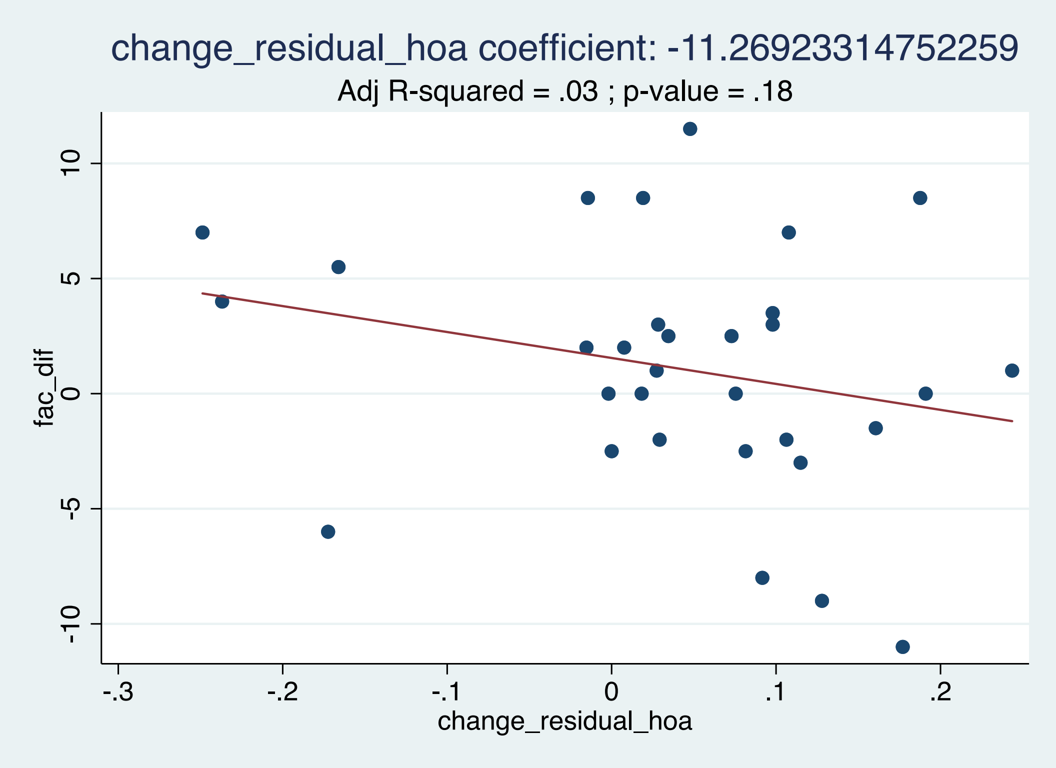
change_residual_hoa coefficient: -11.26923314752259
Adj R-squared = .03 ; p-value = .18
fac_dif
10
5
0
-5
-10
-.3
-.2
-.1
0
.1
.2
change_residual_hoa

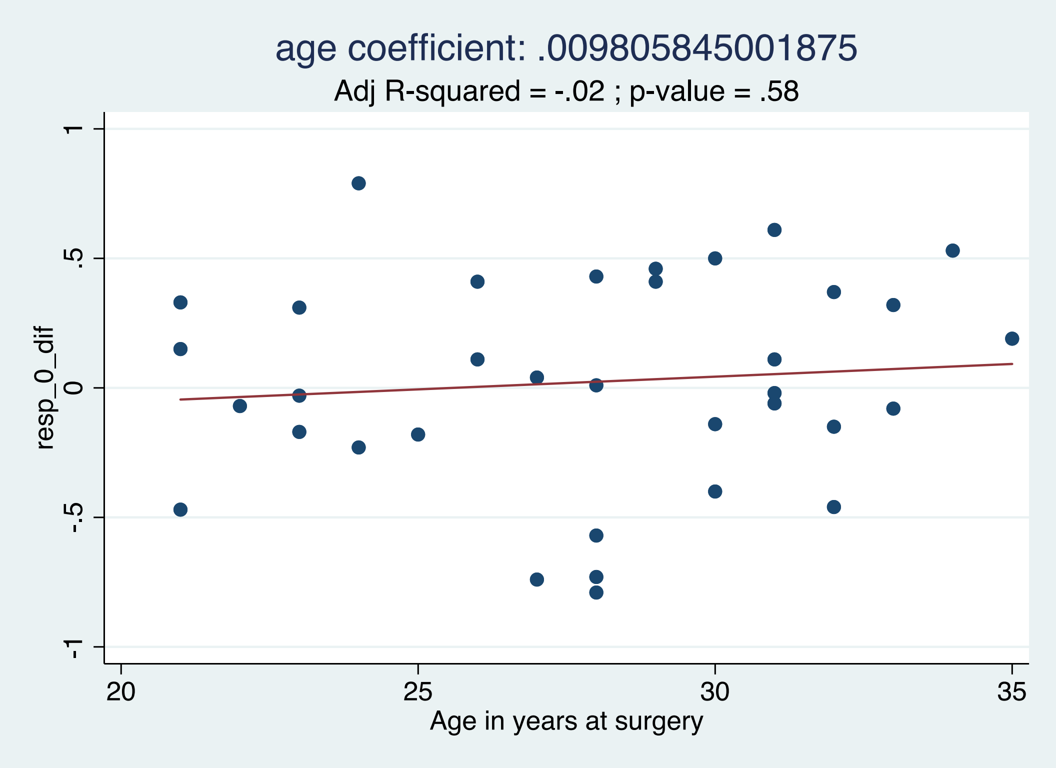
age coefficient: .009805845001875
Adj R-squared = -.02 ; p-value = .58
resp_0_dif
1
.5
0
-.5
-1
20
25
30
35
Age in years at surgery

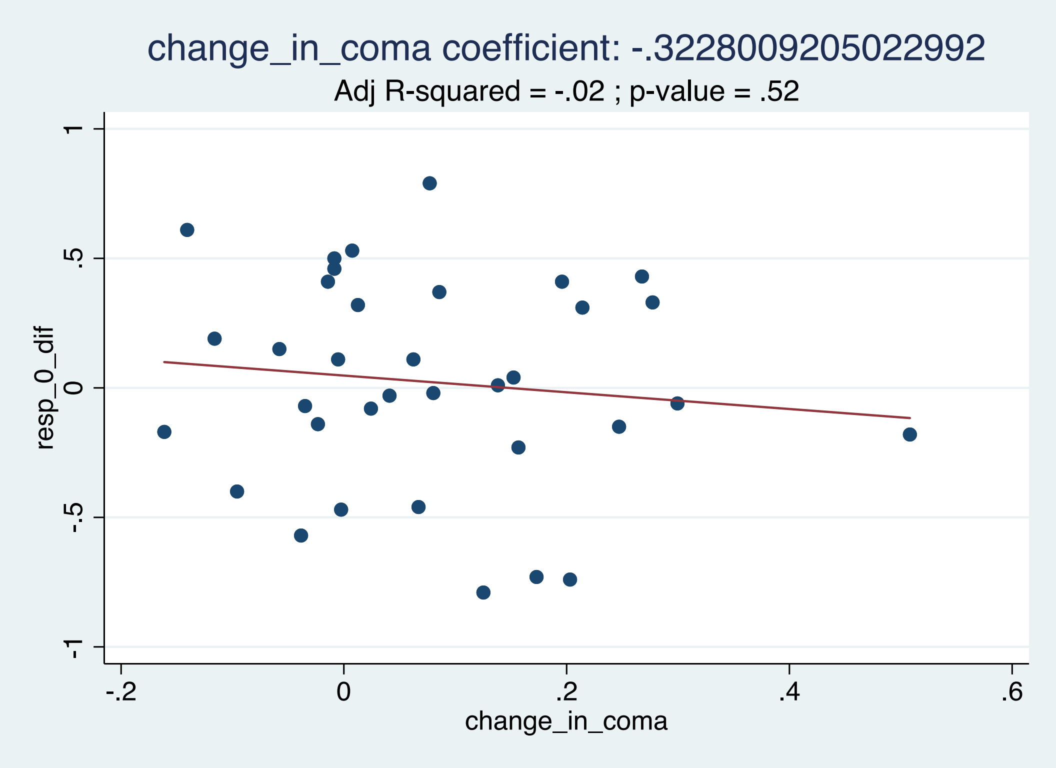
change_in_coma coefficient: -.32280092050229920
Adj R-squared = -.02 ; p-value = .52
resp_0_dif
1
.5
0
-.5
-1
-.2
0
.2
.4
.6
change_in_coma

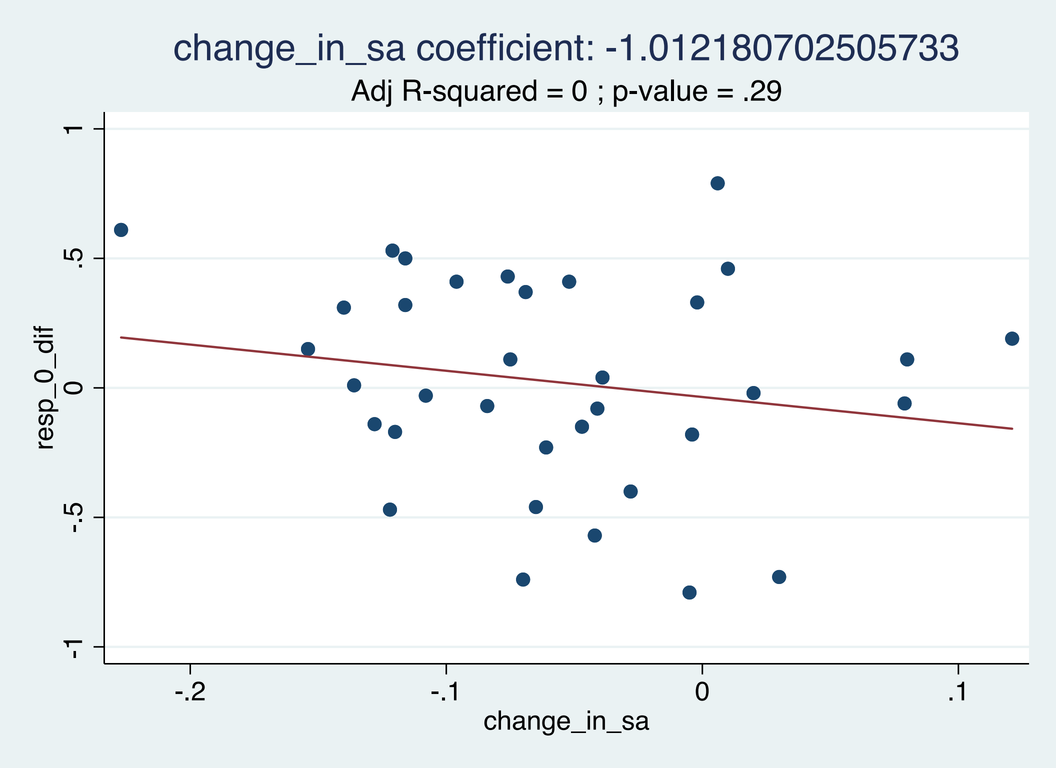
change_in_sa coefficient: -1.012180702505733
Adj R-squared = 0 ; p-value = .29
resp_0_dif
1
.5
0
-.5
-1
-.2
-.1
0
.1
change_in_sa

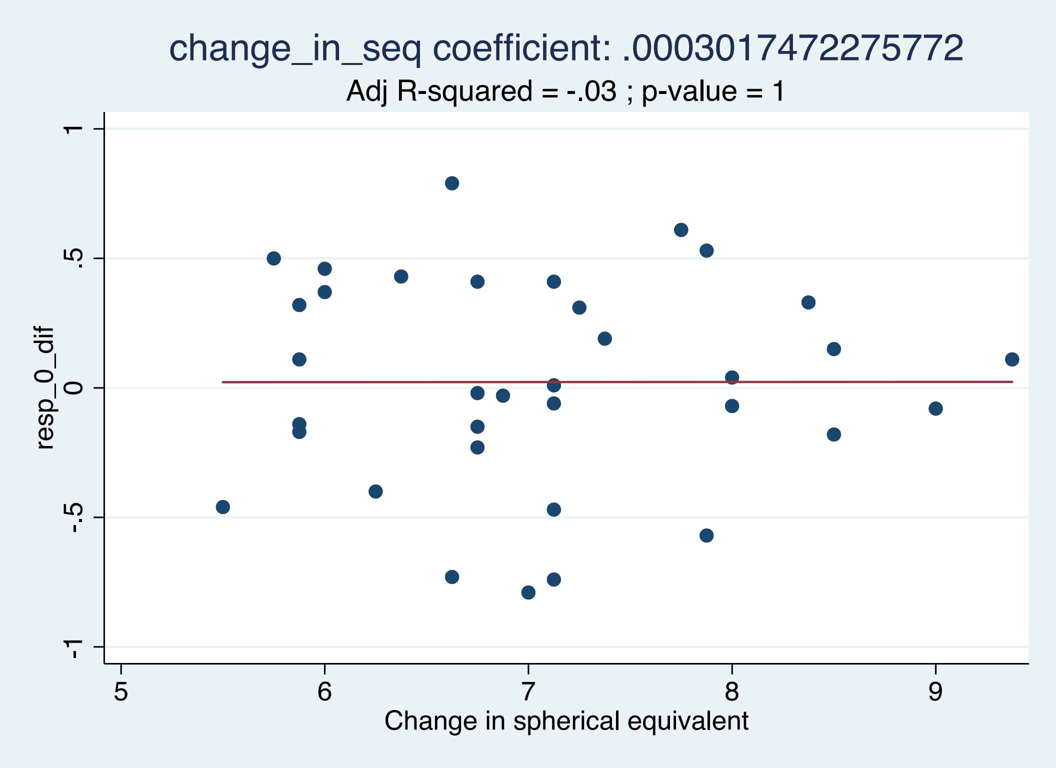
change_in_seq coefficient: .00030174722755772
Adj R-squared = -.03 ; p-value = 1
resp_0_dif
1
.5
0
-.5
-1
5
6
7
8
9
Change in spherical equivalent

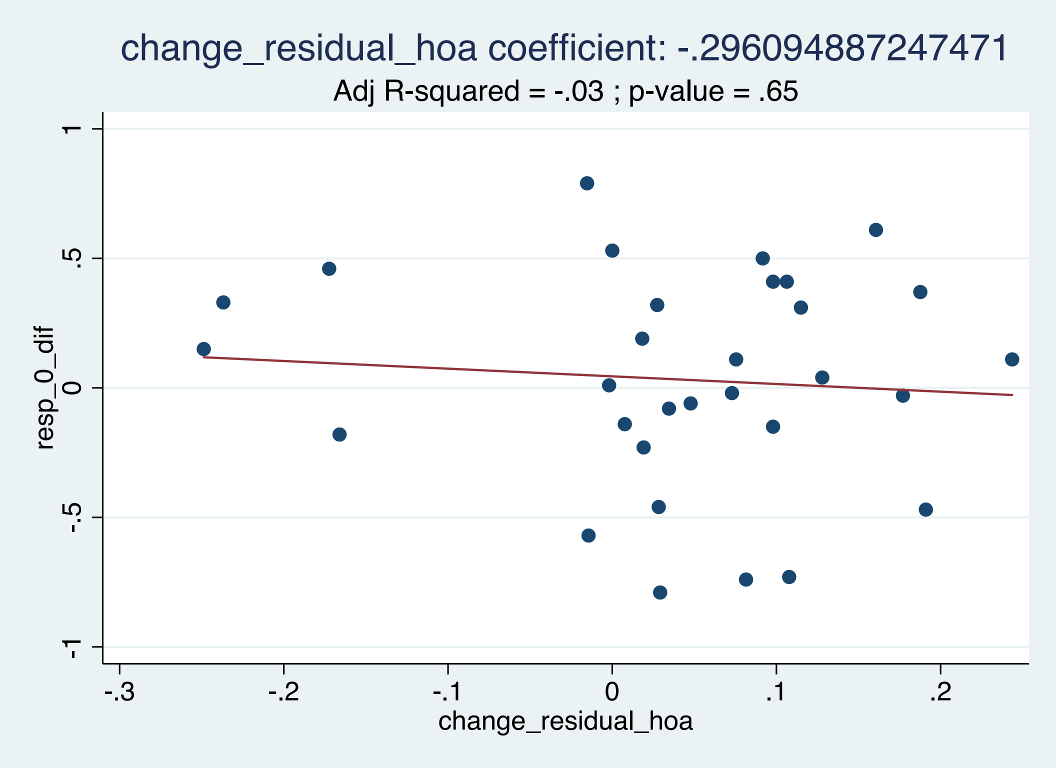
change_residual_hoa coefficient: -.296094887247471
Adj R-squared = -.03 ; p-value = .65
resp_0_dif
1
.5
0
-.5
-1
-.3
-.2
-.1
0
.1
.2
change_residual_hoa

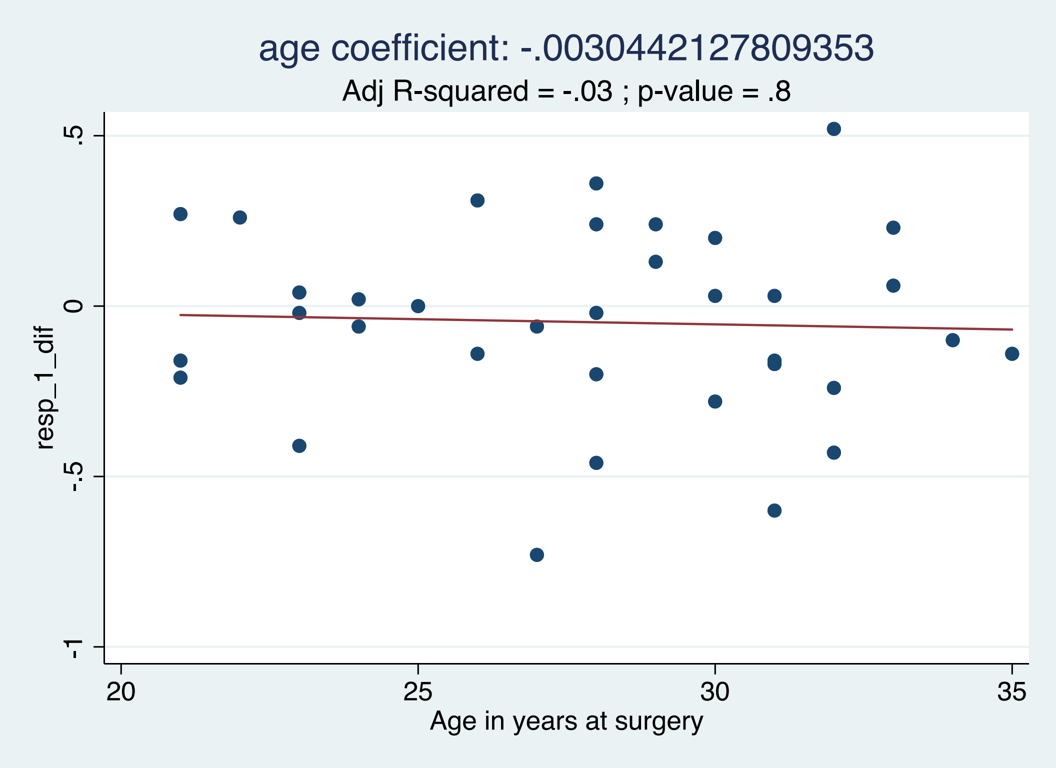
age coefficient: -.0030442127809353
Adj R-squared = -.03 ; p-value = .8
resp_1_dif
.5
0
-.5
-1
20
25
30
35
Age in years at surgery

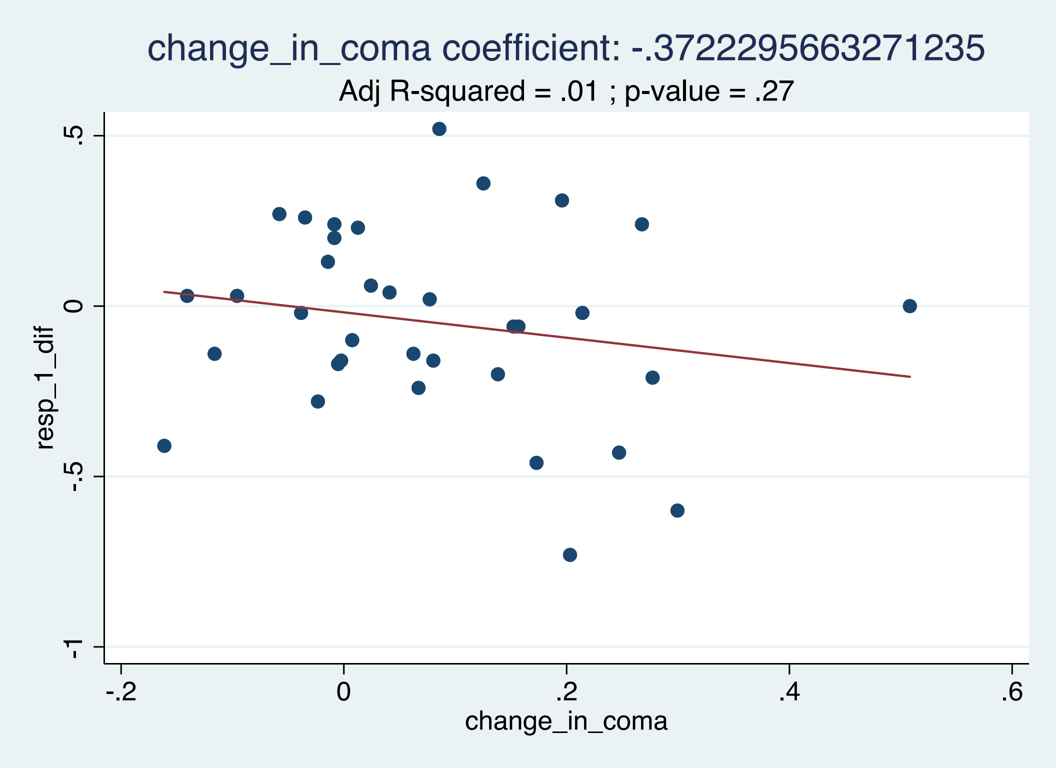
change_in_coma coefficient: -.3722295663271235
Adj R-squared = .01 ; p-value = .27
resp_1_dif
.5
0
-.5
-1
-.2
0
.2
.4
.6
change_in_coma

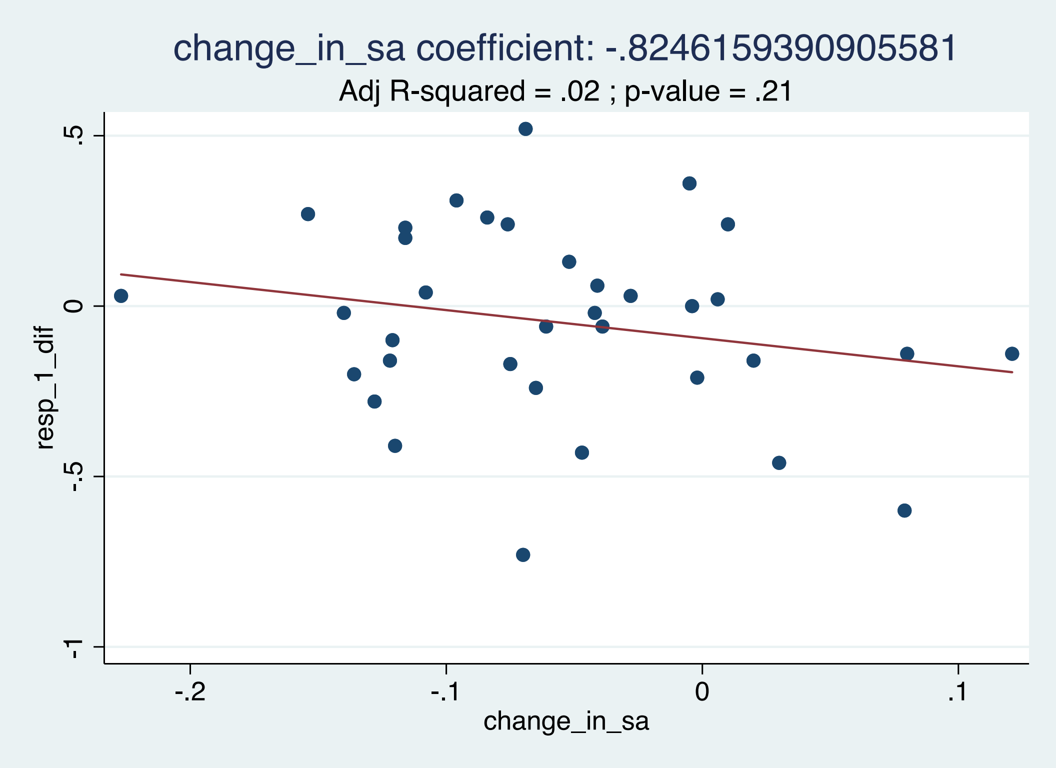
change_in_sa coefficient: -.82461593909055\81
Adj R-squared = .02 ; p-value = .21
resp_1_dif
.5
0
-.5
-1
-.2
-.1
0
.1
change_in_sa

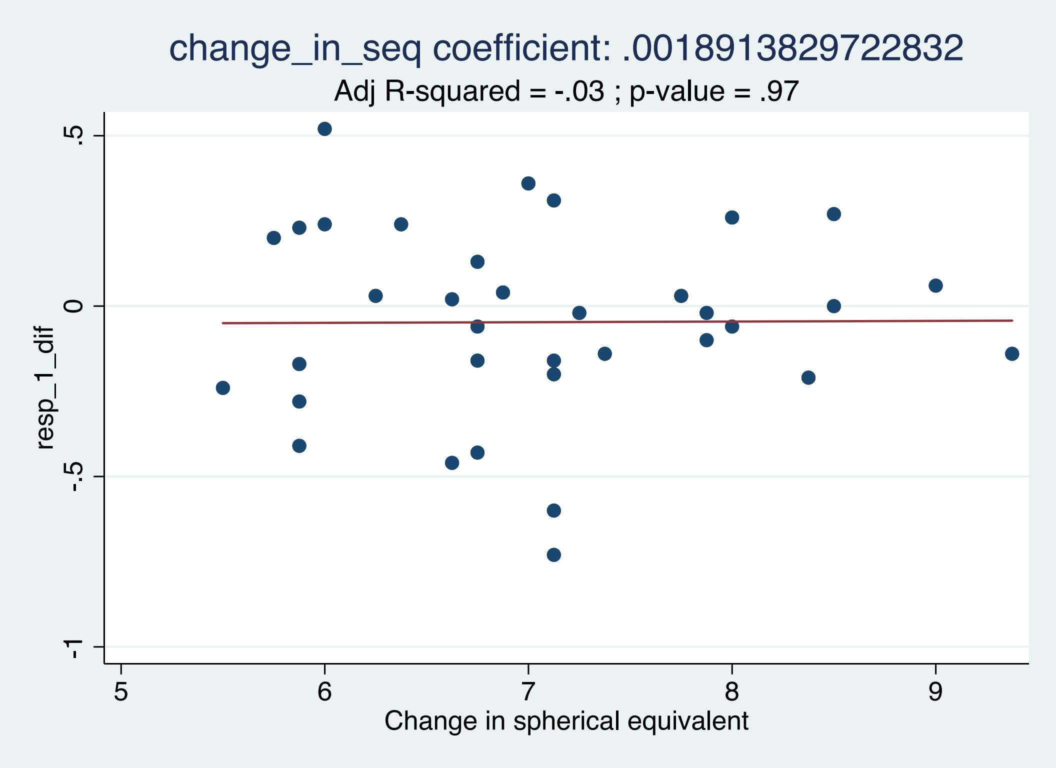
change_in_seq coefficient: .0018913829722832
Adj R-squared = -.03 ; p-value = .97
resp_1_dif
-1
-.5
0
.5
5
6
7
8
9
Change in spherical equivalent

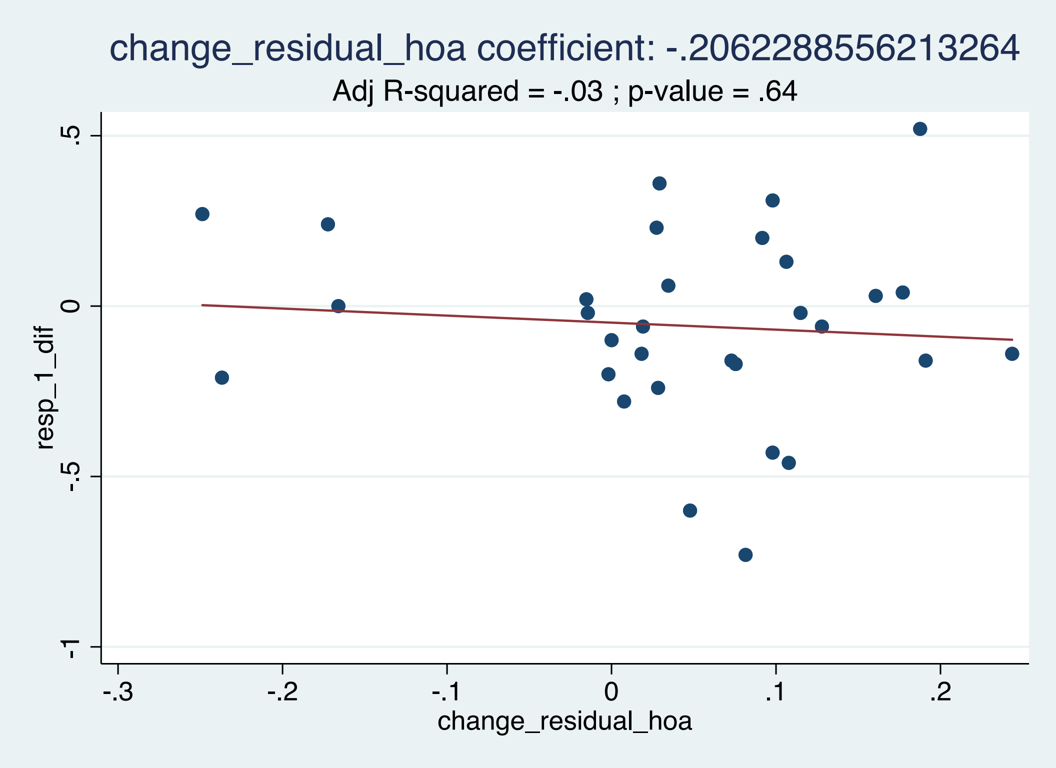
change_residual_hoa coefficient: -.2062288556213264
Adj R-squared = -.03 ; p-value = .64
resp_1_dif
.5
0
-.5
-1
-.3
-.2
-.1
0
.1
.2
change_residual_hoa

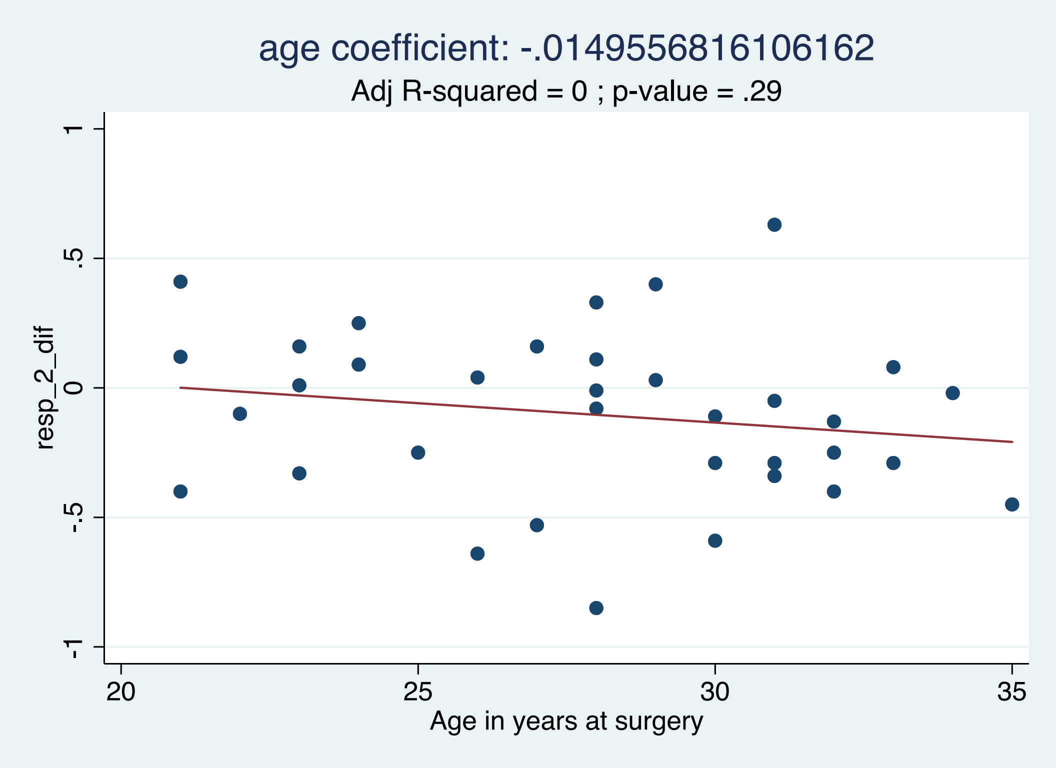

age coefficient: -.0149556816106162
Adj R-squared = 0 ; p-value = .29
1
.5
0
-.5
-1
resp_2_dif
20
25
30
35
Age in years at surgery

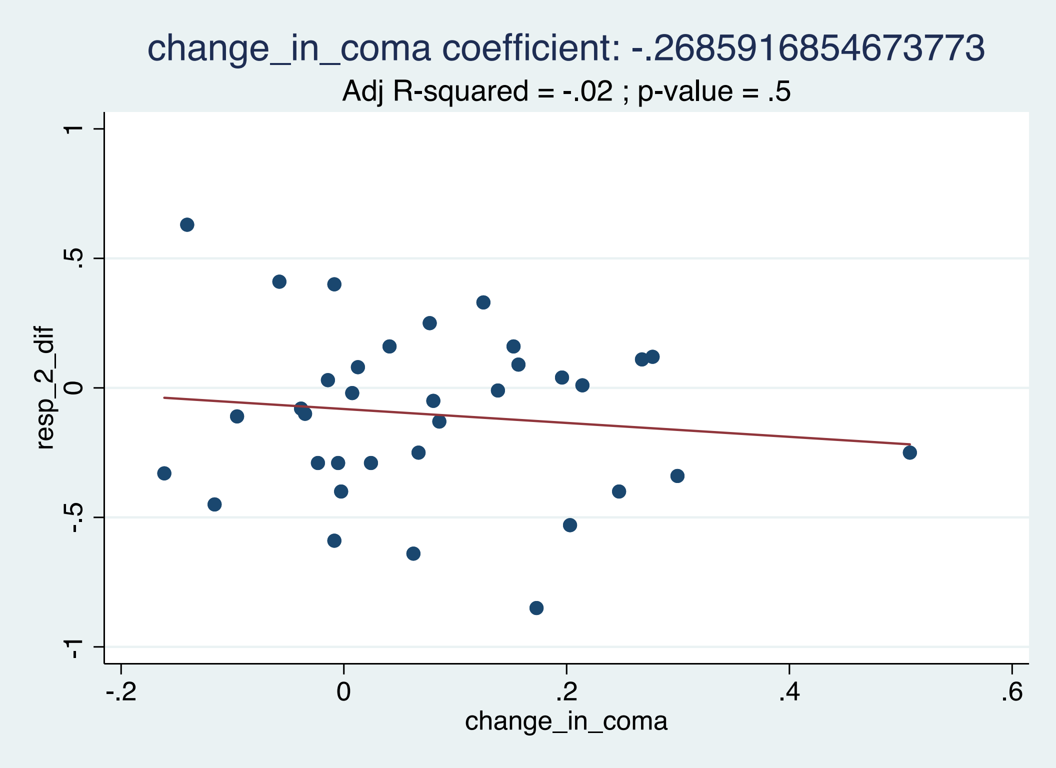
change_in_coma coefficient: -.26859168546737733
Adj R-squared = -.02 ; p-value = .5
resp_2_dif
1
.5
0
-.5
-1
-.2
0
.2
.4
.6
change_in_coma

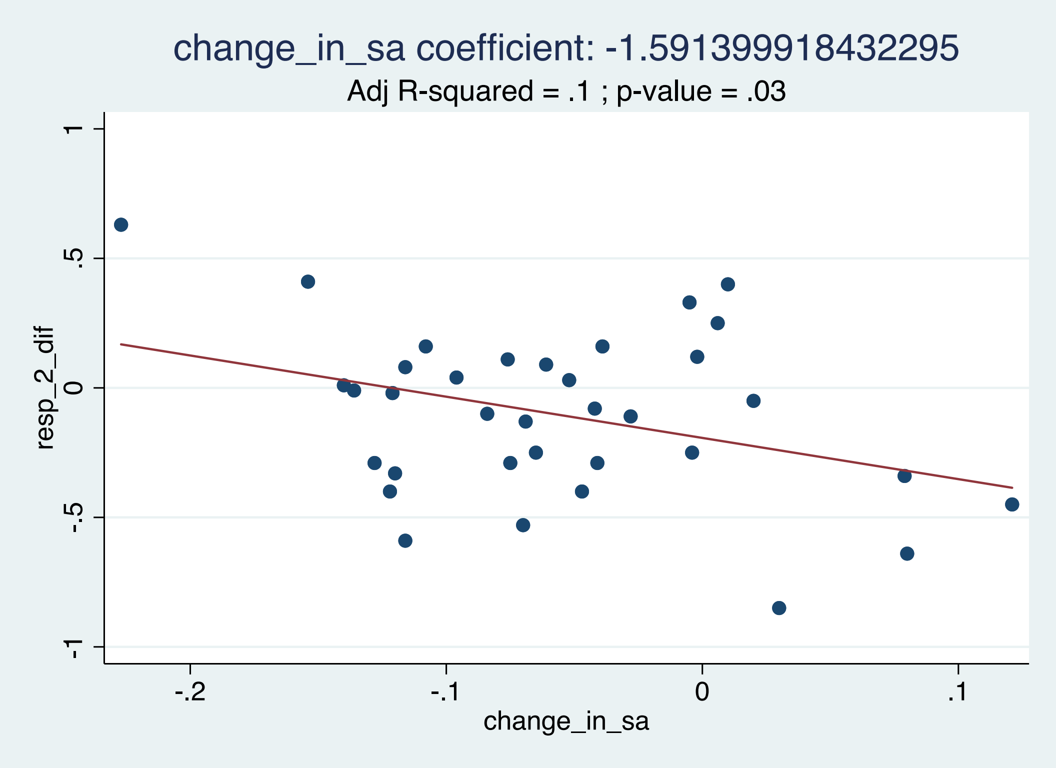
change_in_sa coefficient: -1.591399918432295
Adj R-squared = .1 ; p-value = .03
resp_2_dif
1
.5
0
-.5
-1
-.2
-.1
0
.1
change_in_sa

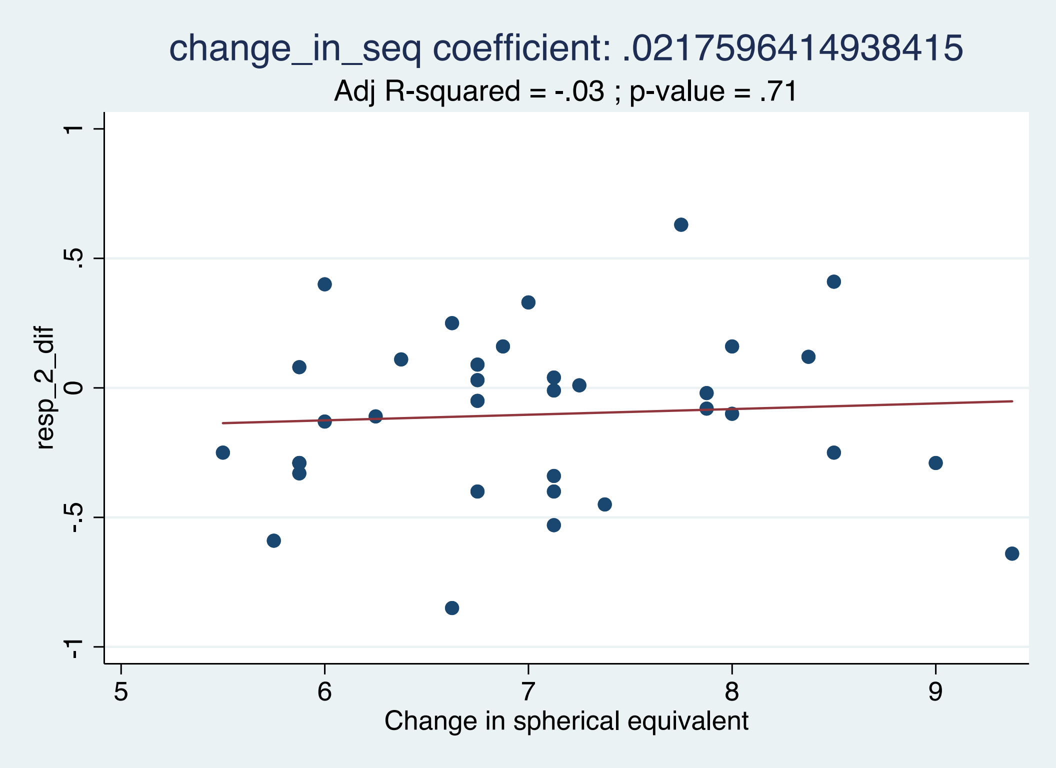
change_in_seq coefficient: .0217596414938415
Adj R-squared = -.03 ; p-value = .71
resp_2_dif
1
.5
0
-.5
-1
5
6
7
8
9
Change in spherical equivalent

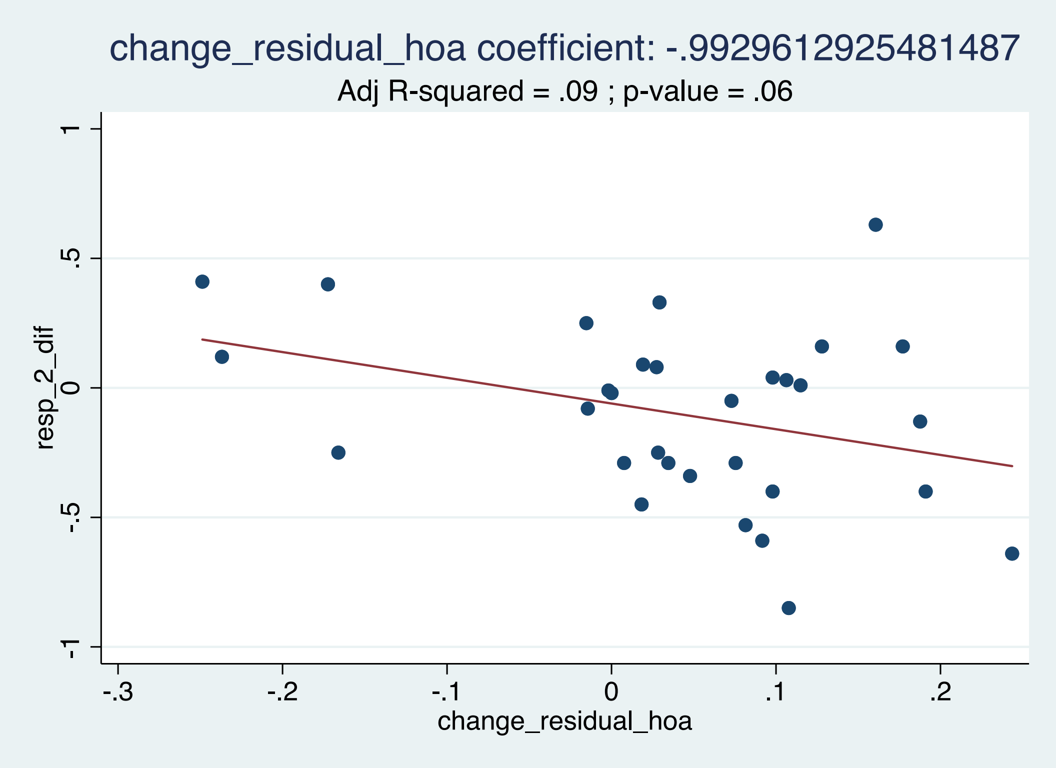
change_residual_hoa coefficient: -.99296129254814870
Adj R-squared = .09 ; p-value = .06
resp_2_dif
1
.5
0
-.5
-1
-.3
-.2
-.1
0
.1
.2
change_residual_hoa

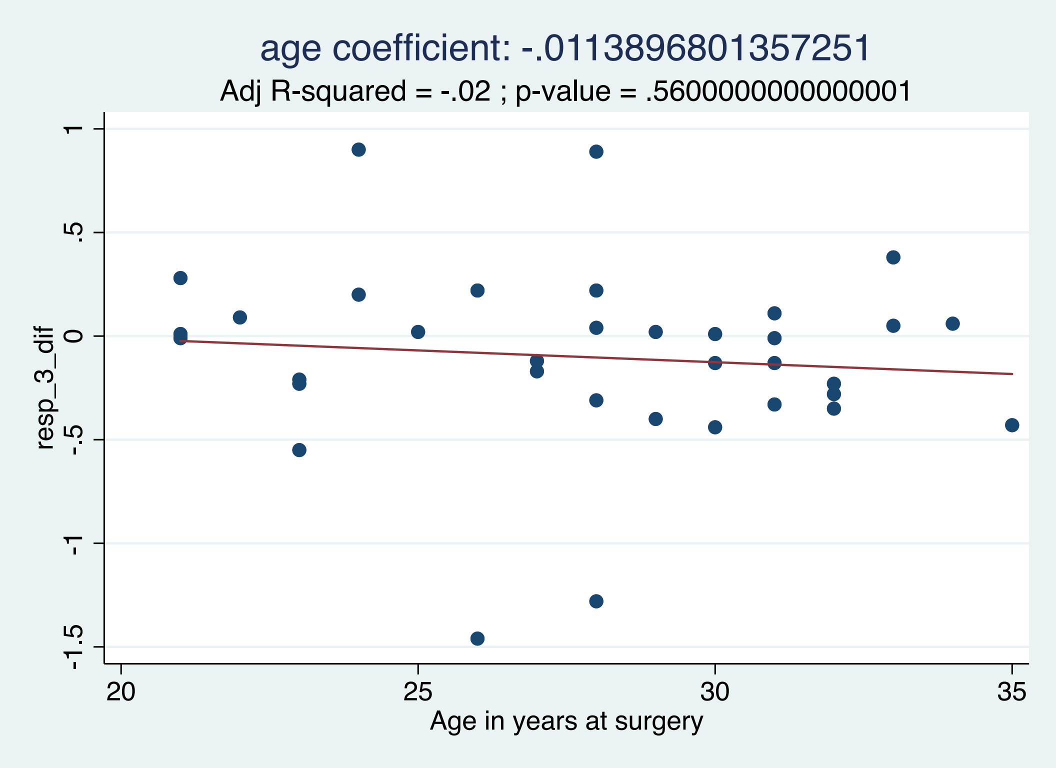
age coefficient: -.0113896801357251
Adj R-squared = -.02 ; p-value = .5600000000000001
resp_3_dif
Age in years at surgery
1
.5
0
-.5
-1
-1.5
20
25
30
35

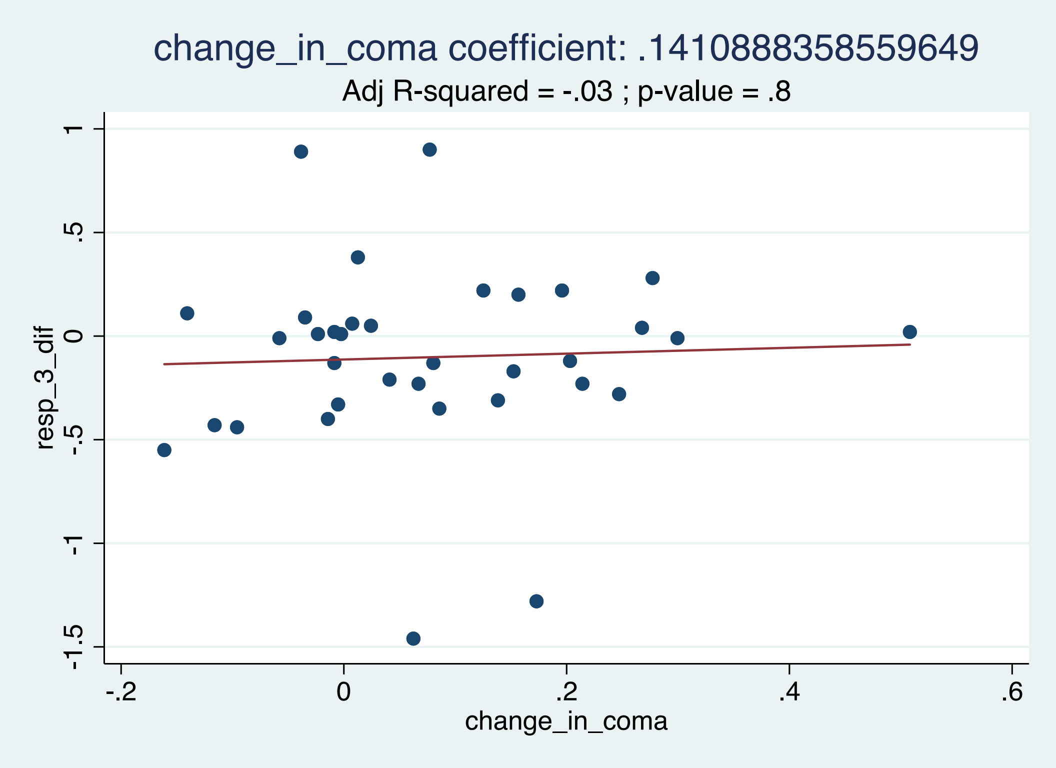
change_in_coma coefficient: .1410888358559649
Adj R-squared = -.03 ; p-value = .8
resp_3_dif
1
.5
0
-.5
-1
-1.5
-.2
0
.2
.4
.6
change_in_coma

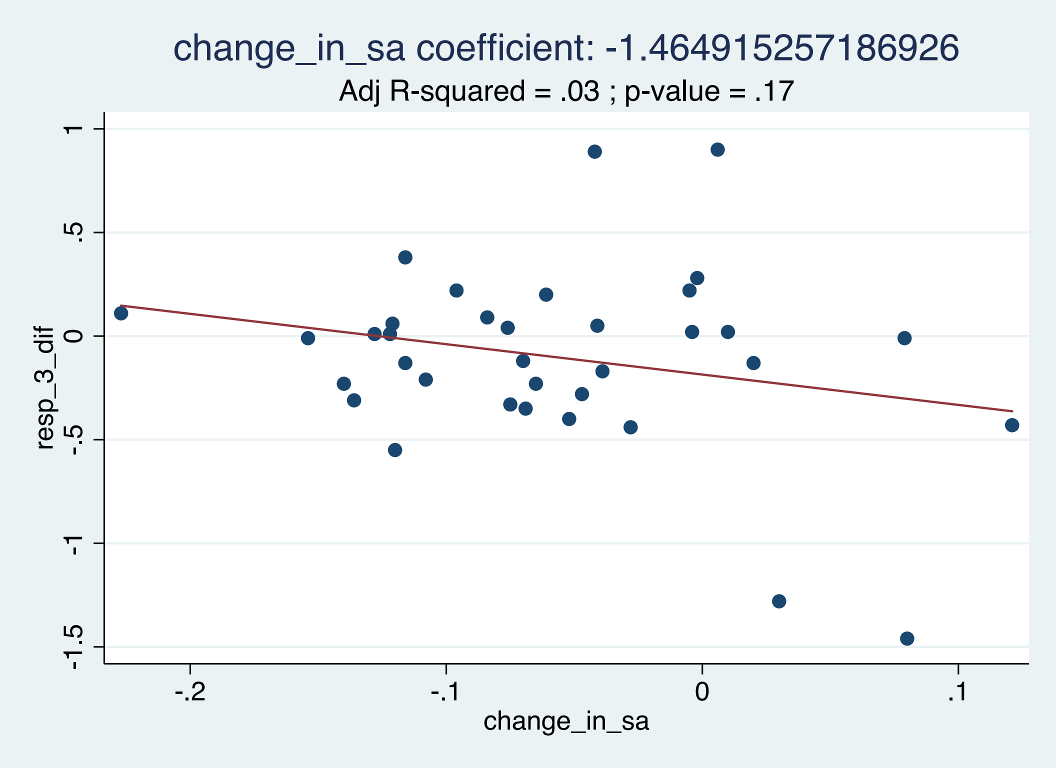

change_in_sa coefficient: -1.464915257186926
Adj R-squared = .03 ; p-value = .17
resp_3_dif
1
.5
0
-.5
-1
-1.5
-.2
-.1
0
.1
change_in_sa

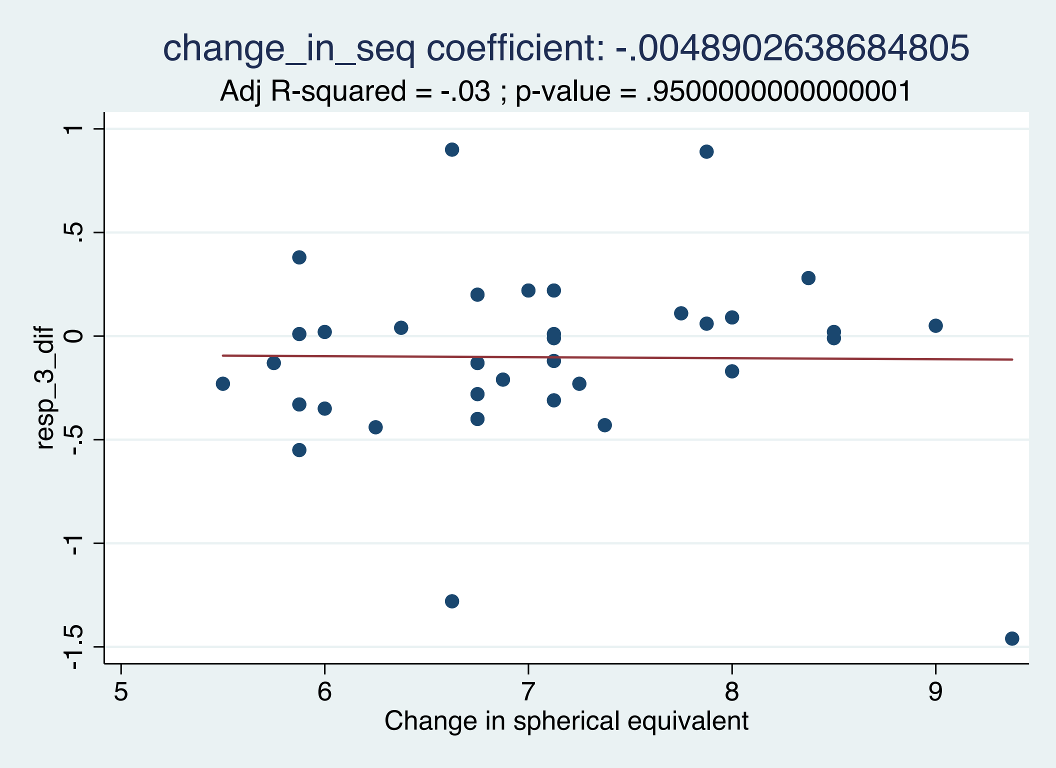

change_in_seq coefficient: -.0048902638684805
Adj R-squared = -.03 ; p-value = .9500000000000001
resp_3_dif
1
.5
0
-.5
-1
-1.5
5
6
7
8
9
Change in spherical equivalent

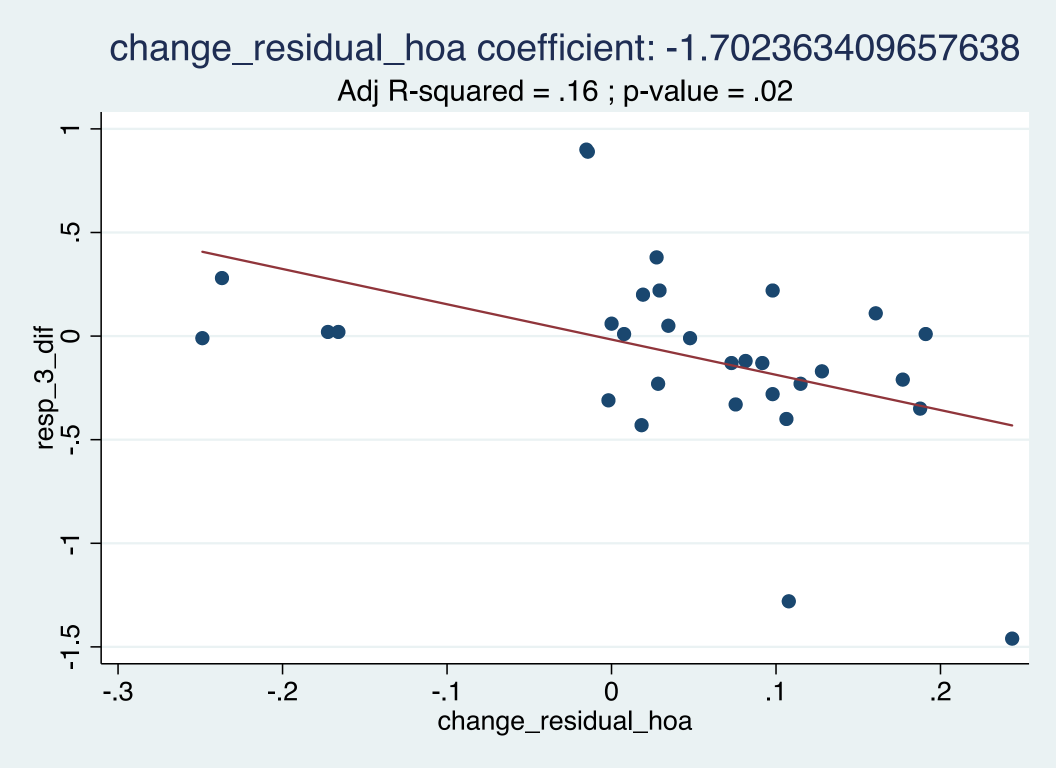
change_residual_hoa coefficient: -1.702363409657638
Adj R-squared = .16 ; p-value = .02
resp_3_dif
1
.5
0
-.5
-1
-1.5
-.3
-.2
-.1
0
.1
.2
change_residual_hoa

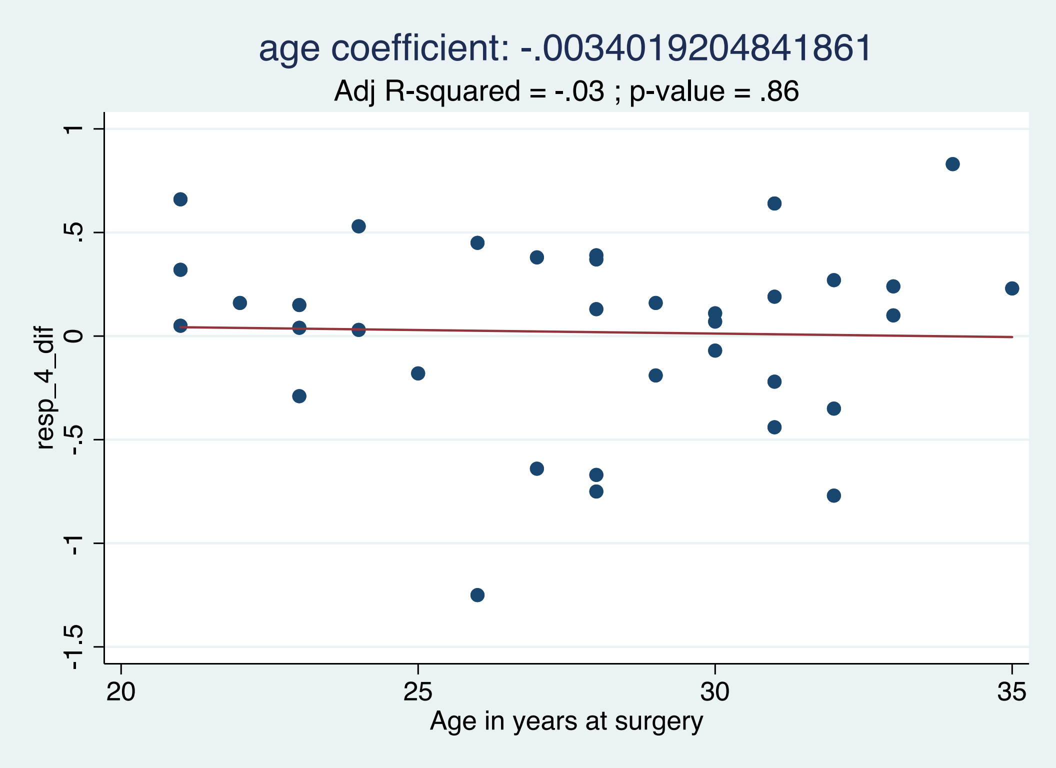
age coefficient: -.0034019204841861
Adj R-squared = -.03 ; p-value = .86
resp_4_dif
1
.5
0
-.5
-1
-1.5
20
25
30
35
Age in years at surgery

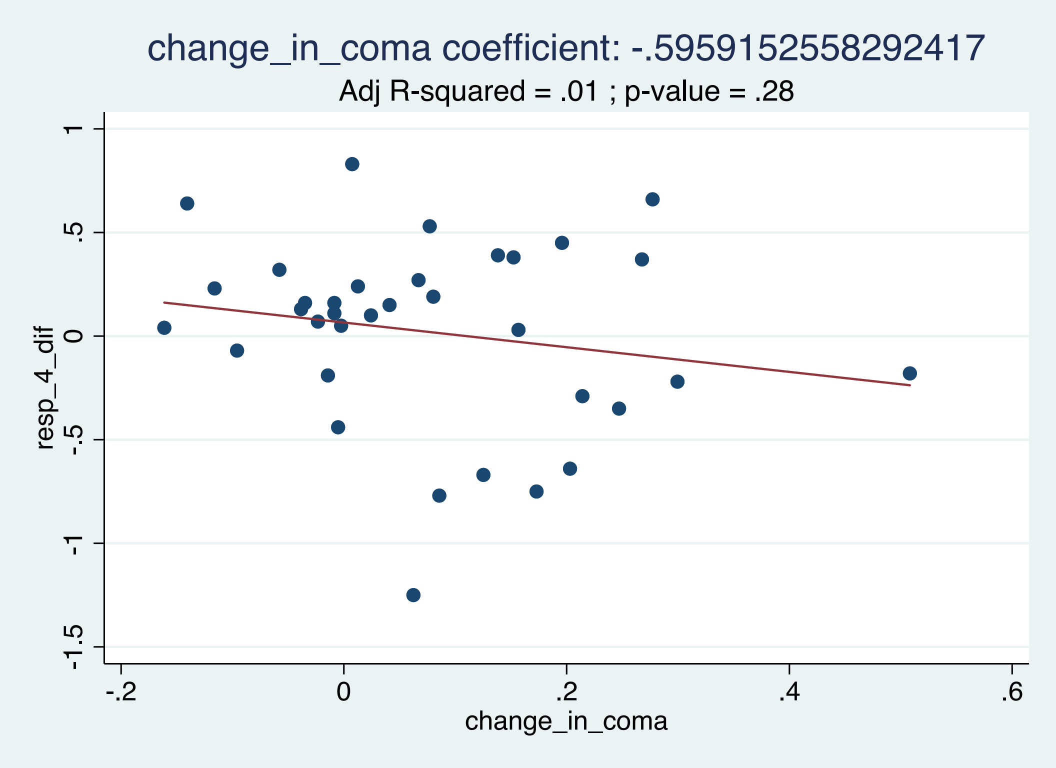
change_in_coma coefficient: -.5959152558292417
Adj R-squared = .01 ; p-value = .28
resp_4_dif
1
.5
0
-.5
-1
-1.5
-.2
0
.2
.4
.6
change_in_coma

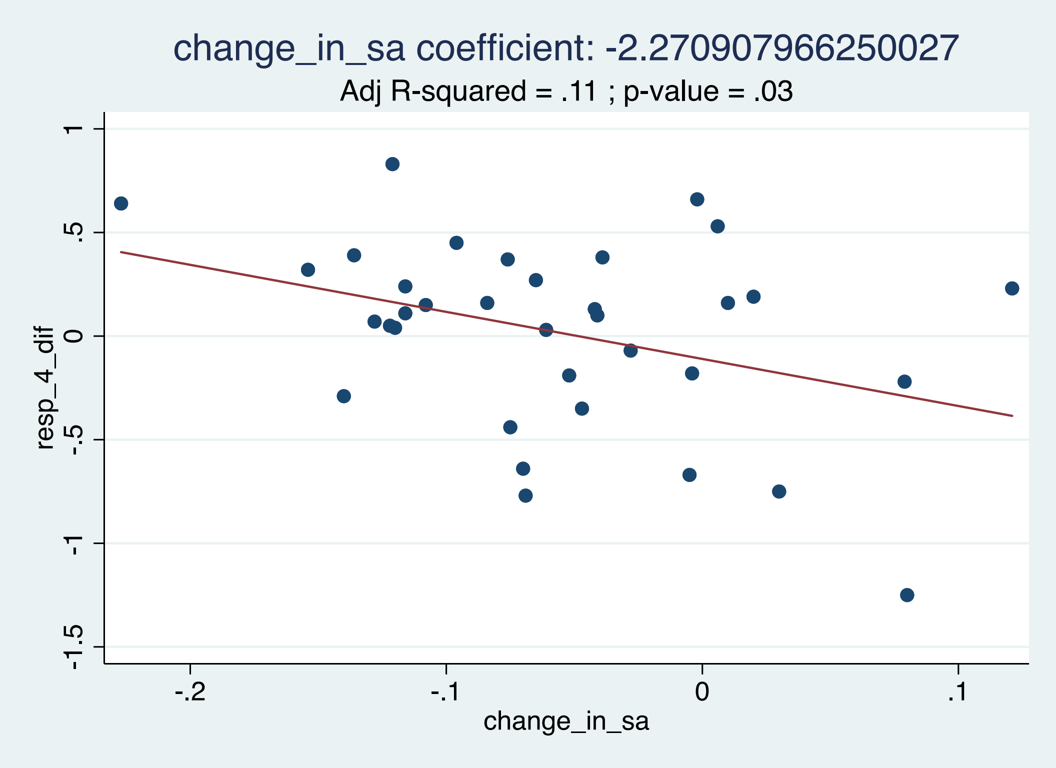
change_in_sa coefficient: -2.270907966250027
Adj R-squared = .11 ; p-value = .03
resp_4_dif
1
.5
0
-.5
-1
-1.5
-.2
-.1
0
.1
change_in_sa

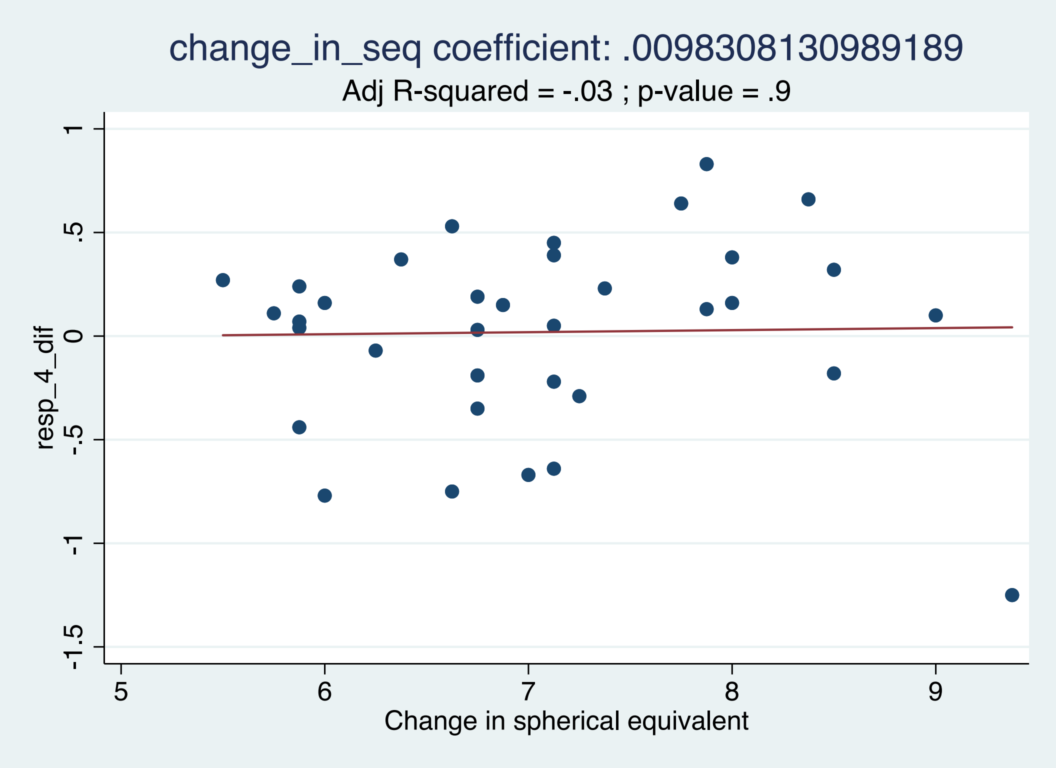

change_in_seq coefficient: .0098308130989189
Adj R-squared = -.03 ; p-value = .9
resp_4_dif
1
.5
0
-.5
-1
-1.5
5
6
7
8
9
Change in spherical equivalent

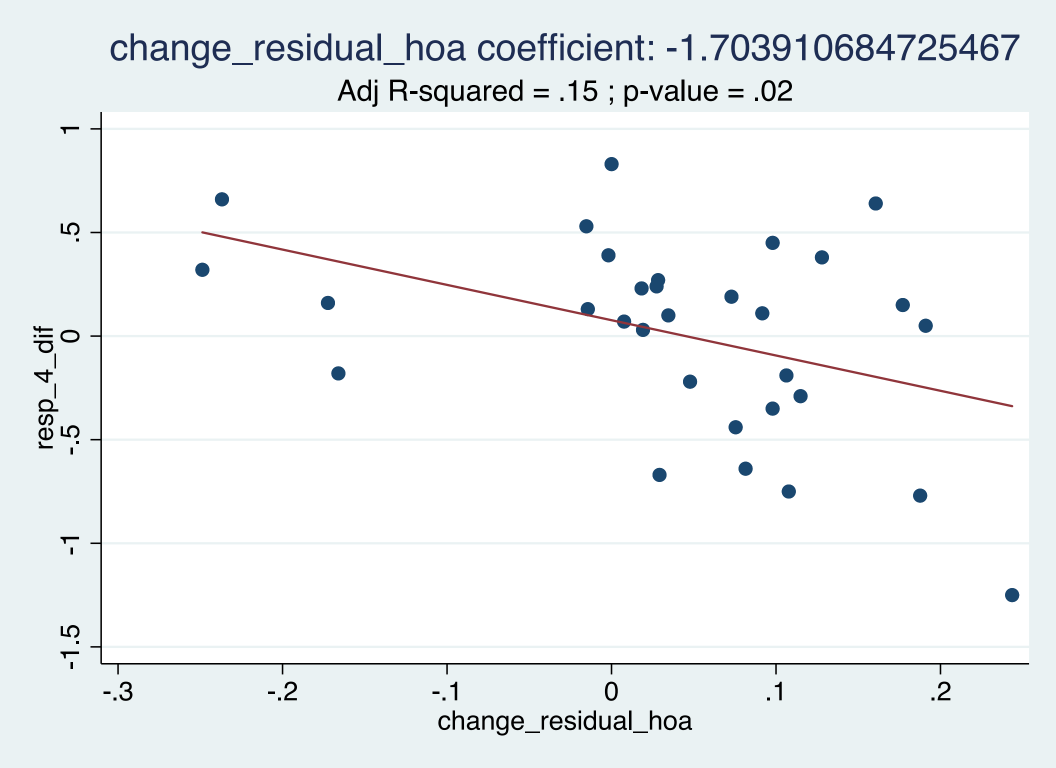
change_residual_hoa coefficient: -1.703910684725467
Adj R-squared = .15 ; p-value = .02
resp_4_dif
1
.5
0
-.5
-1
-1.5
-.3
-.2
-.1
0
.1
.2
change_residual_hoa

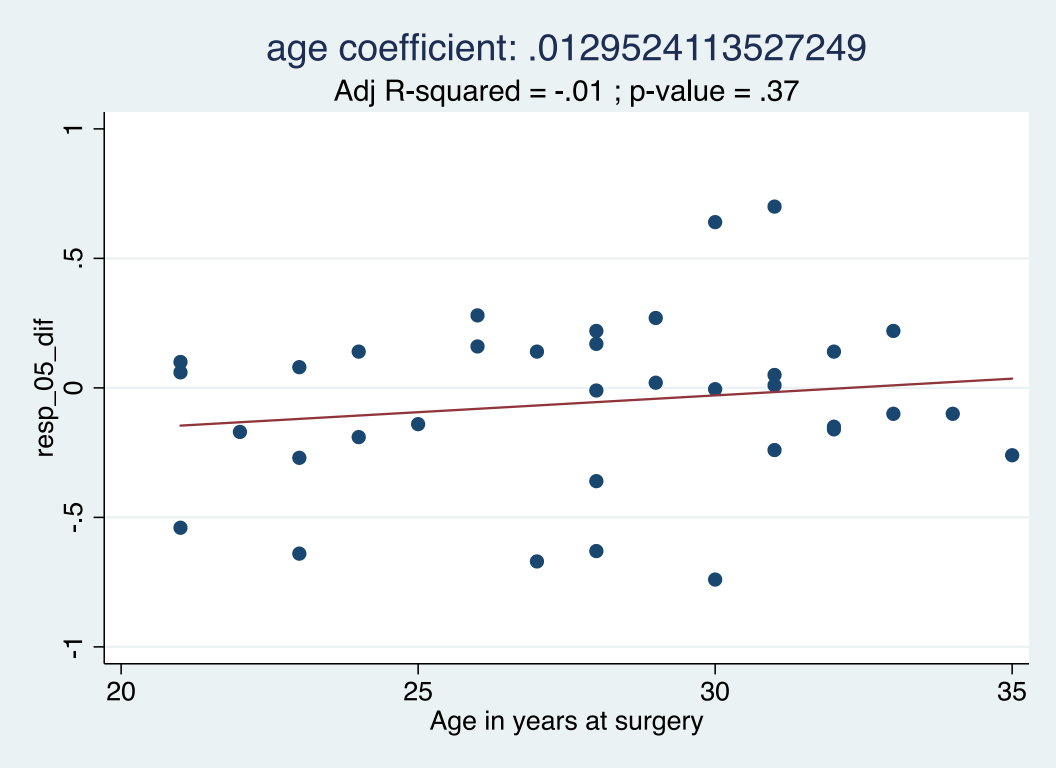
age coefficient: .0129524113527249
Adj R-squared = -.01 ; p-value = .37
resp_05_dif
1
.5
0
-.5
-1
20
25
30
35
Age in years at surgery

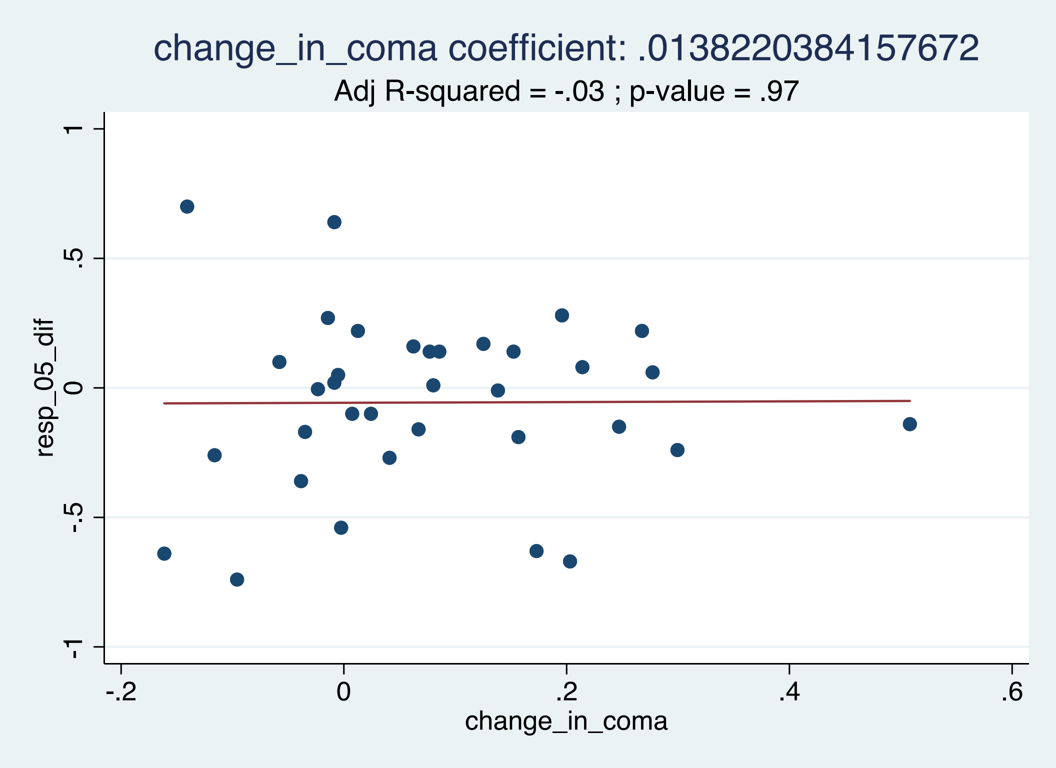
change_in_coma coefficient: .0138220384157672
Adj R-squared = -.03 ; p-value = .97
resp_05_dif
1
.5
0
-.5
-1
-.2
0
.2
.4
.6
change_in_coma

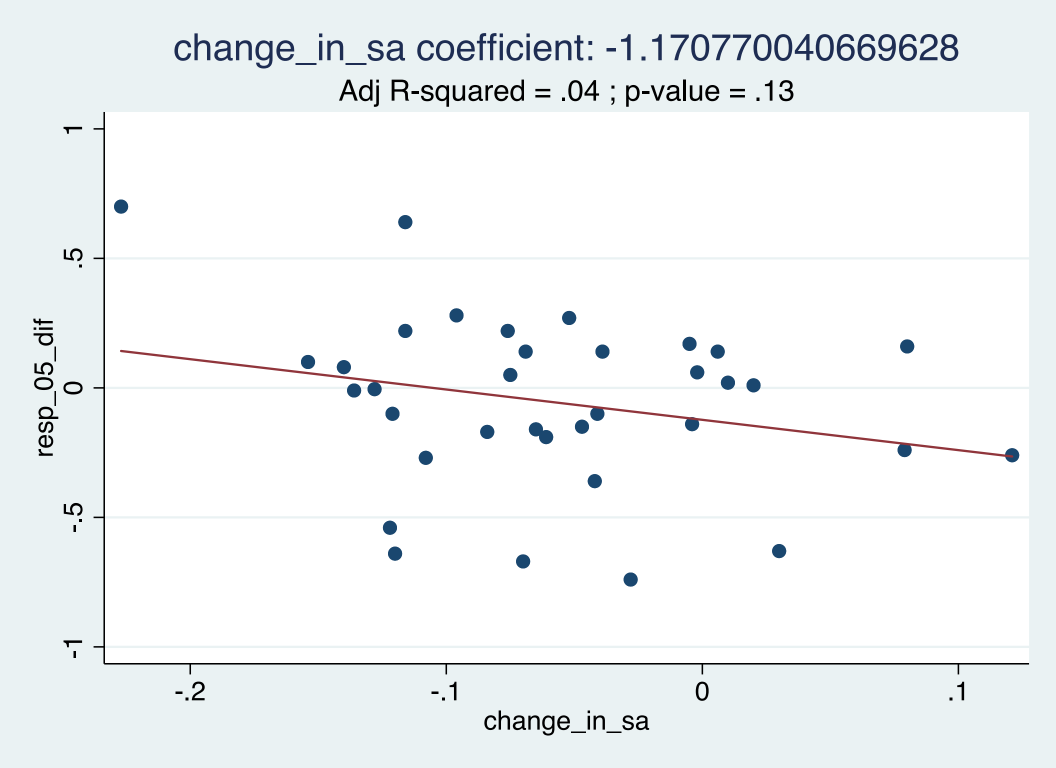
change_in_sa coefficient: -1.170770040669628
Adj R-squared = .04 ; p-value = .13
resp_05_dif
1
.5
0
-.5
-1
-.2
-.1
0
.1
change_in_sa

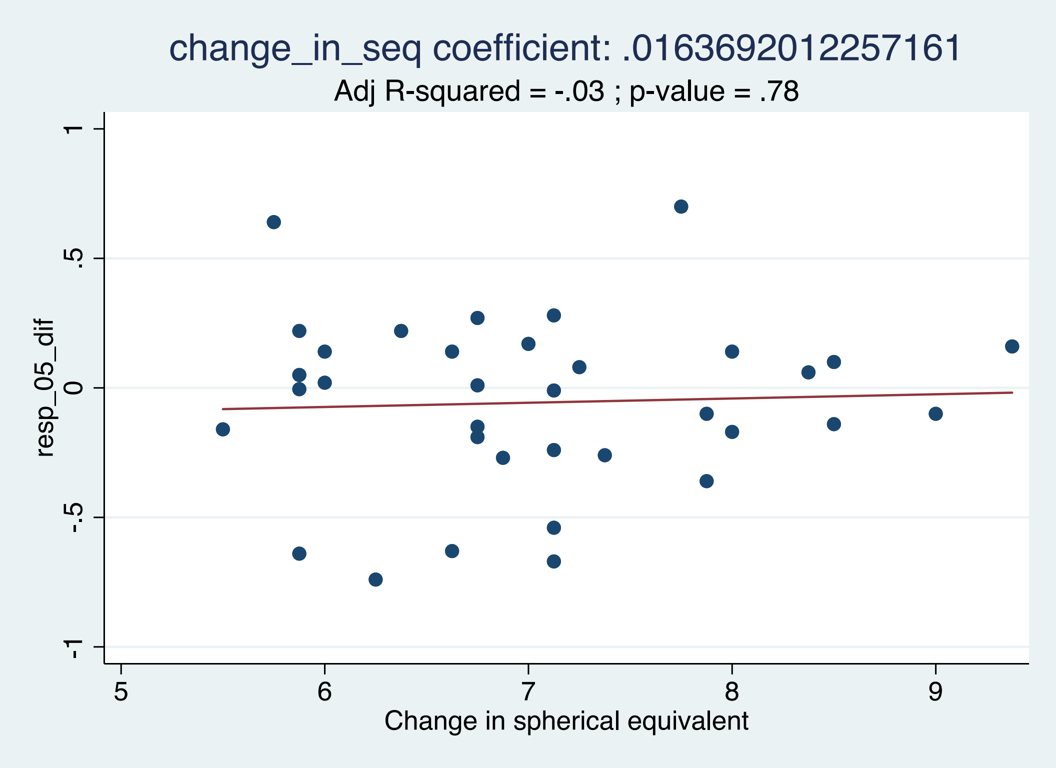
change_in_seq coefficient: .0163692012257161
Adj R-squared = -.03 ; p-value = .78
resp_05_dif
1
.5
0
-.5
-1
5
6
7
8
9
Change in spherical equivalent

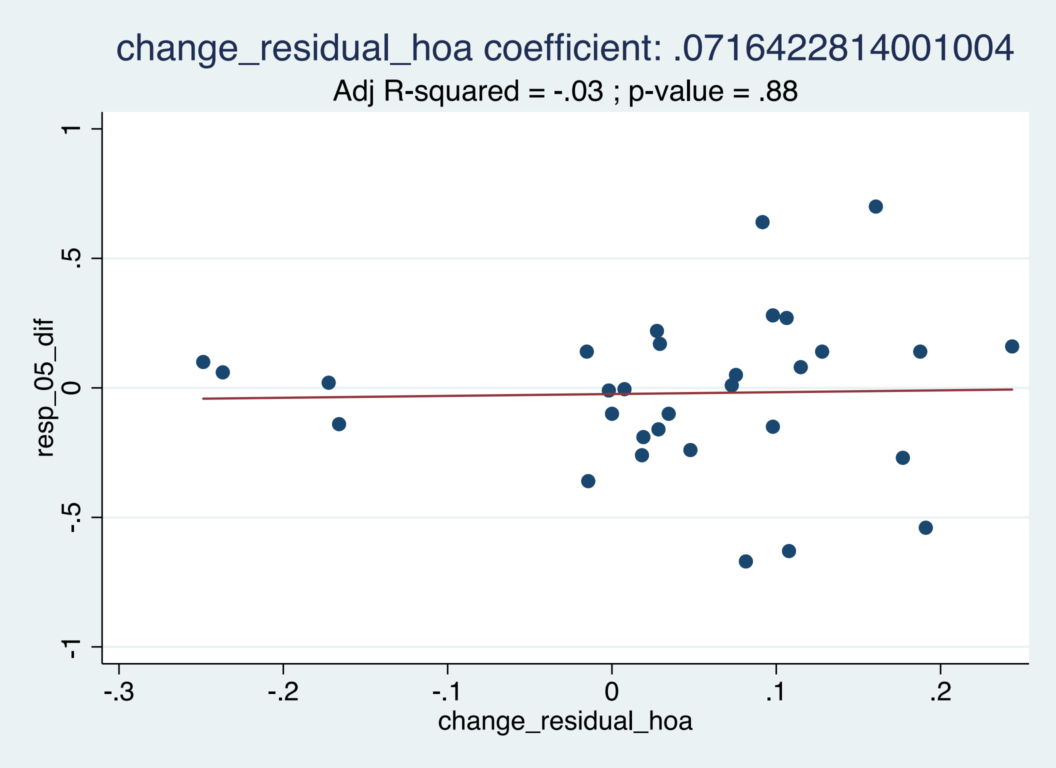

change_residual_hoa coefficient: .0716422814001004
Adj R-squared = -.03 ; p-value = .88
resp_05_dif
1
.5
0
-.5
-1
-.3
-.2
-.1
0
.1
.2
change_residual_hoa
